# Supplementary material for: On-liquid surface synthesis of diyne-linked two-dimensional polymer crystals
Source: Nat Commun. 2025 Sep 8;16:8243. doi: 10.1038/s41467-025-63768-6 (PMC12417544; doi:10.1038/s41467-025-63768-6)
Supplement: Supplementary file 1 — Supplementary Information [file 41467_2025_63768_MOESM1_ESM.pdf]

Supplementary Information

On-Liquid Surface Synthesis of Diyne-linked Two-Dimensional Polymer Crystals

Ye Yang[1], Yufeng Wu[2], Chang Liu[3,4], Mike Hambsch[5], Tiange Dong[2], David Bodesheim[6], Mahabir Prasad[7,8], Arezoo Dianat[6], Thomas D. Kühne[7,8,9], Gianaurelio Cuniberti[6,10], Stefan C. B. Mannsfeld[5], Stuart S. P. Parkin[2], Renhao Dong[11,12], Zhiyong Wang[1,2]*, and Xinliang Feng[1,2]*

[1]Center for Advancing Electronics Dresden & Faculty of Chemistry and Food Chemistry, Technische Universität Dresden, Dresden, Germany.

[2]Max Planck Institute for Microstructure Physics, Halle (Saale), Germany.

[3]MOE Engineering Research Center of Membrane and Water Treatment, and Key Lab of Adsorption and Separation Materials & Technologies of Zhejiang Province, Department of Polymer Science and Engineering, Zhejiang University, Hangzhou, China.

[4]The "Belt and Road" Sino-Portugal Joint Lab on Advanced Materials, International Research Center for X Polymers, Zhejiang University, Hangzhou, China.

[5]Center for Advancing Electronics Dresden & Faculty of Electrical and Computer Engineering, Technische Universität Dresden, Dresden, Germany.

[6]Institute for Materials Science and Max Bergmann Center for Biomaterials, TUD Dresden University of Technology, Dresden, Germany.

[7]Center for Advanced Systems Understanding, Görlitz, Germany.

[8]Helmholtz-Zentrum Dresden-Rossendorf, Dresden, Germany.

[9]Institute of Artificial Intelligence, Chair of Computational System Sciences, Technische Universität Dresden, Dresden, Germany.

[10]Dresden Center for Computational Materials Science (DCMS), TUD Dresden University of Technology, Dresden, Germany.

[11]Department of Chemistry, The University of Hong Kong, Hong Kong, China.

[12]Materials Innovation Institute for Life Sciences and Energy (MILES), HKU-SIRI, Shenzhen, China.

*E-mail: zhiyong.wang@mail.mpi-halle.mpg.de; xinliang.feng@mpi-halle.mpg.de

Table of Contents

Supplementary Methods 4
Supplementary Fig. 1 8
Supplementary Fig. 2 9
Supplementary Fig. 3 10
Supplementary Fig. 4 11
Supplementary Fig. 5 12
Supplementary Fig. 6 13
Supplementary Fig. 7 14
Supplementary Fig. 8 15
Supplementary Fig. 9 16
Supplementary Fig. 10 17
Supplementary Fig. 11 18
Supplementary Fig. 12 20
Supplementary Fig. 13 21
Supplementary Fig. 14 22
Supplementary Fig. 15 23
Supplementary Fig. 16 24
Supplementary Fig. 17 25
Supplementary Fig. 18 26
Supplementary Fig. 19 27
Supplementary Fig. 20 28
Supplementary Fig. 21 29
Supplementary Fig. 22 30
Supplementary Fig. 23 31
Supplementary Fig. 24 33
Supplementary Fig. 25 34
Supplementary Fig. 26 35
Supplementary Fig. 27 36
Supplementary Fig. 28 37
Supplementary Fig. 29 38
Supplementary Fig. 30 39
Supplementary Fig. 31 40

Supplementary Fig. 32 41
Supplementary Fig. 33 42
Supplementary Fig. 34 43
Supplementary Fig. 35 44
Supplementary Fig. 36 45
Supplementary Fig. 37 46
Supplementary Fig. 38 47
Supplementary Fig. 39 48
Supplementary Fig. 40 49
Supplementary Fig. 41 50
Supplementary Fig. 42 51
Supplementary Fig. 43 52
Supplementary Fig. 44 53
Supplementary Fig. 45 54
Supplementary Fig. 46 55
Supplementary Fig. 47 56
Supplementary Fig. 48 57
Supplementary Fig. 49 58
Supplementary Fig. 50 59
Supplementary Fig. 51 60
Supplementary Fig. 52 61
Supplementary Fig. 53 62
Supplementary Fig. 54 63
Supplementary Fig. 55 64
Supplementary Fig. 56 65
Supplementary Table 1 66
Supplementary Table 2 67
Supplementary Table 3 68
Supplementary References 69

Supplementary Methods

Materials. 5,10,15,20-tetrakis(4-ethynylphenyl)porphyrin (TEPP) and 1,2,3,4,5,6-hexakis((trimethylsilyl)-ethynyl)benzene (HEB-TMS) were purchased from Jilin Chinese Academy of Sciences - Yanshen Technology Co., Ltd. (China). Copper-derived TEPP and 5-(bromo)-10,15,20-(triphenyl)porphyrin were obtained from PorphyChem (France). Perfluorooctadecanoic acid (PFS) was purchased from Apollo Scientific Ltd. (UK). Phenylacetylene, tetrakis(triphenylphosphine)palladium(0) ($Pd(PPh_3)_4$), CuCl and pyridine were purchased from Sigma-Aldrich, GmbH (Germany). Trimethyl((4-(4,4,5,5-tetramethyl-1,3,2-dioxaborolan-2-yl)phenyl)ethynyl)silane was obtained from BLD Pharm Deutschland, GmbH (Germany). All compounds were used as received.

Silicon substrates (1 cm×1 cm) for scanning electron microscopy (SEM). SiO_2 (300 nm)/Si substrates (1 cm×1 cm) were used for optical microscopy (OM) and atomic force microscopy (AFM) measurements. Gold-coated silicon wafer from Sigma-Aldrich was used for Fourier-transform infrared spectroscopy (FTIR), Raman spectroscopy, and X-ray photoelectron spectroscopy (XPS) tests. Quartz plate from Plano GmbH, Germany, (1.5 cm×1.5 cm, 1 mm thickness) was used for UV-Vis absorption spectroscopy investigations. Copper meshes from Plano GmbH were used for the TEM measurements.

Substrates cleaning. The cleanness of substrates is crucial in surface science. The surface of the Silicon substrates, SiO_2(300 nm)/Si substrates, and Quartz plates substrates were cleaned with a fresh piranha solution (80 vol.% H_2SO_4:20 vol.% H_2O_2 30 % aqueous solution) for 1 h. Then, 100 ml Milli-Q water and 100 ml isopropanol was utilized to wash the surface under sonication, respectively.

Synthesis of 5,10,15-triphenyl-20-(4-((trimethylsilyl)ethynyl)phenyl)porphyrin. 5-(bromo)-10,15,20-(triphenyl)porphyrin (25 mg, 0.04 mmol), Trimethyl((4-(4,4,5,5-tetramethyl-1,3,2-dioxaborolan-2-yl)phenyl)ethynyl)silane (18.23 mg, 0.06 mmol), K_2CO_3 (33.57 mg, 0.24 mmol) and $Pd(PPh_3)_4$ (4.68 mg, 0.004 mmol) were added into a mixture of toluene/ethanol/water (2/1/1), and heated at 100 °C for 24 hours of reflux. The solvent was removed in vacuo and the residue was purified by column chromatography (hexane/DCM 1:1, v/v) to afford the purple solid (17.6 mg, 0.025 mmol, 62%). ^1H NMR (300 MHz, $CDCl_3$): 8.89-8.80 (d, 8H), 8.24-8.18 (d, 8H), 7.90-7.85 (d, 3H), 7.79-7.72 (d, 8H). The obtained compound was mixed with tetra-n-butylammonium fluoride (TBAF) to remove the TMS, and then used for the model reaction.

Synthesis procedure of Model I. 40 ml of DMAc-Milli-Q water mixture (v:v = 1:1) was added into

a crystallization dish (diameter, 6 cm). Then, 28 µl of PFS solution (1 mg ml^{-1} in $CHCl_3$) was spread on the DMAc-H_2O surface using a micropipette to form a stable and crystalline surfactant monolayer. After 30 min, 1 ml of a mixture of CuCl (0.03 mmol) and pyridine (0.06 mmol) aqueous solution was gently injected into the liquid subphase using a syringe. After 2 h, the reaction was initiated by adding 1 ml of 10-(4-ethynylphenyl)-5,15,20-triphenylporphyrin (**1**) (4.2 µmol, in DMAc). The reactions were then kept undisturbed at 1 °C for 24 h, yielding macroscopic organic films on the DMAc-H_2O surface. To obtain the products on the DMAc-H_2O surface, the films were transferred onto the SiO_2/Si substrate via a horizontal dipping approach, followed by vacuum drying at 80 °C (Supplementary Figs. 9b,c). To isolate the bulk-phase products, 2 ml of the subphase liquid was extracted from the bottom of the crystallization dish using a syringe and dried under vacuum at 80 °C, yielding a solid residue deposited on the inner wall of the vial (Supplementary Figs. 9a,b). The collected materials were subsequently re-dissolved in $CHCl_3$ for matrix-assisted laser desorption/ionization-time-of-flight mass spectrometry.

Synthesis procedure of Model II. 40 ml of DMAc-Milli-Q water mixture (v:v = 1:1) was added into a crystallization dish (diameter, 6 cm). Then, 28 µl of PFS solution (1 mg ml^{-1} in $CHCl_3$) was spread on the DMAc-H_2O surface using a micropipette to form a stable and crystalline surfactant monolayer. After 30 min, 1 ml of a mixture of CuCl (0.03 mmol) and pyridine (0.06 mmol) aqueous solution was gently injected into the liquid subphase using a syringe. After 2 h, the reaction was initiated by adding 1 ml of phenylacetylene (**3**) (98.0 µmol in DMAc). The reactions were then kept undisturbed at 1 °C for 24 h, yielding macroscopic organic films on the DMAc-H_2O surface. To obtain the products on the DMAc-H_2O surface, the films were transferred onto the SiO_2/Si substrate via a horizontal dipping approach, followed by vacuum drying at 80 °C (Supplementary Figs. 9b,c). To isolate the bulk-phase products, 2 ml of the subphase liquid was extracted from the bottom of the crystallization dish using a syringe and dried under vacuum at 80 °C, yielding a solid residue deposited on the inner wall of the vial (Supplementary Figs. 9a,b). The collected materials were subsequently re-dissolved in $CDCl_3$ for nuclear magnetic resonance measurement.

Characterizations. ^{1}H NMR spectra were performed at 30 °C on a Bruker AV-II 300 spectrometer. The high-resolution mass spectrometry analysis of products in model reactions was performed on a Bruker Autoflex Speed matrix-assisted laser desorption/ionization-time-of-flight mass spectrometry (MALDI TOF MS, Bruker Daltonics, Bremen, Germany) with the linear model using trans-2-[3-(4-tert-butylphenyl)-2-methyl-2-propenylidene]malononitrile (DCTB) as matrix. The surface tensions of the liquids were measured using a Force Tensiometer K100 (KRÜSS Scientific). Attenuated total reflection-Fourier transform infrared (FTIR) spectra were recorded by using a Bruker Optics ALPHA-

E spectrometer equipped with Attenuated Total Reflectance (ATR) module. A Cary 5000 UV-vis-near infrared (NIR) Spectrophotometer was used to test the adsorption spectra. XPS spectra were obtained by using an X-ray photoelectron spectroscopy (XPS, XSAM800, Kratos Analytical, UK). Optical microscopy (OM, Zeiss) and atomic force microscopy (AFM, NT-MDT) were used to measure the morphology and thickness of 2DPs. X-ray diffraction (XRD) patterns were obtained on an X-ray diffractometer (Aeris Research Edition, Malvern Panalytical Company) using Cu-Kα radiation (λ = 0.15418 nm) at 40 kV and 15 mA at room temperature in reflection geometry. High-resolution transmission electron microscopy (HRTEM) was conducted using JEOL JEM F200 operated at 200 kV acceleration voltage equipped with a GATAN OneView CMOS camera for fast imaging. Scanning electron microscopy (SEM, Zeiss Gemini 500) equipped with energy-dispersive X-ray spectroscopy was employed to monitor the morphology.

DFT calculations of energy barriers. The reaction energies of the model compounds on the DMAc-H_2O surface, and in the bulk solution were calculated based on the density functional theory (DFT) method using the CP2K package.[1] The geometry of all the structures in the ground state was optimized with GGA-PBE exchange-correlation functional[2] and Goedecker-Teter-Hutter (GTH) pseudopotentials using dispersion corrections.[3] To define the reaction energies of compounds on the DMAc-H_2O surface (in the DMAc-H_2O bulk), the energy of the DMAc-H_2O slab (bulk) has been subtracted from the total energy of the systems.

DFT calculations of TEPP molecules underneath a PFS monolayer with different angles. The geometry optimization and calculation of energy for differently oriented TEPP molecules underneath a PFS monolayer were performed using CP2K[4]. The system was placed in vacuum at the center of the box of dimension 30×30×70 $Å^3$ to make sure that it does not interact with its mirror image. The box was considered periodic on all three directions. The DFT calculations were performed using the QUICKSTEP module based on the mixed Gaussian and plane waves (GPW) approach[5]. The Kohn-Sham orbitals were described in terms of the DZVP-MOLOPT double-zet basis set[6]. The Goedecker-Teter-Hutter (GTH) pseudopotentials[3,7] were employed for the core electrons and a plane wave cutoff of 400 Ry was employed. The PBE exchange-correlation density functional was chosen in consistent with the basis set and pseudopotential.

In-situ XRF measurement. The in-situ XRF measurement was performed under the total reflection condition, which limits the X-ray penetration depth to ~6 nm[8]. This shallow probing depth ensures that

the detected fluorescence signal predominantly arises from ions localized at or near the interface, minimizing contributions from the bulk solution[9]. The XRF signal was collected using an energy-dispersive detector (Amptek X-123SDD, AMETEK Inc., USA) positioned within the experimental housing at an angle of 3° above the horizontal plane and 90° relative to the incident beam direction (Supplementary Fig. 15). This configuration was chosen to effectively minimize background contributions from Compton and elastic scattering. The X-ray beam used for fluorescence measurements features a spot size of 70 μm ×1,000 μm.

Crystal device fabrication. The DY2DP-Por crystal device was fabricated through electron beam lithography (EBL) and focused ion beam (FIB) at ultrahigh vacuum (Supplementary Fig. 50). The crystals were first diluted and uniformly dispersed onto a clean silicon wafer with predefined alignment marks. A layer of PMMA was then spin-coated onto the wafer, serving as the electron beam resist. Crystal device fabrication was identified using an optical microscope. EBL at 30 kV was employed to pattern the selected crystals. After development, the exposed regions were subjected to magnetron sputtering to sequentially deposit a 5 nm titanium (Ti) adhesion layer followed by 50 nm of gold (Au). The unwanted resist and overlying metal were then removed using a standard lift-off process, resulting in the desired electrode pattern. As the height of the single crystals exceeded that of the deposited metal electrodes, direct electrical contact could not be achieved. To establish a reliable connection, platinum (Pt) was deposited at the contact interface between the crystals and the electrodes using FIB deposition, thereby securing the crystals and completing the electrical circuit.

For the fabrication of GDY crystal device, crystals were uniformly dispersed onto a clean silicon substrate with alignment marks. Due to the substantial height of GDY crystals, conventional spin-coated photoresist could not effectively cover them. To overcome this limitation, Pt contacts were first fabricated directly onto the crystal surfaces using FIB deposition. These Pt structures were engineered with a thick region in contact with the crystal to ensure mechanical robustness, and a thin extension (~20 nm) directed outward to interface with the subsequent Ti/Au electrodes. Following Pt deposition, a positive-tone photoresist was spin-coated onto the sample. Electrode patterns were defined using the Maskless Aligner MLA 150. After development, a 5 nm Ti adhesion layer and 50 nm of Au were deposited by magnetron sputtering. Finally, a standard lift-off process was used to remove the residual photoresist and excess metal.

Supplementary Figures

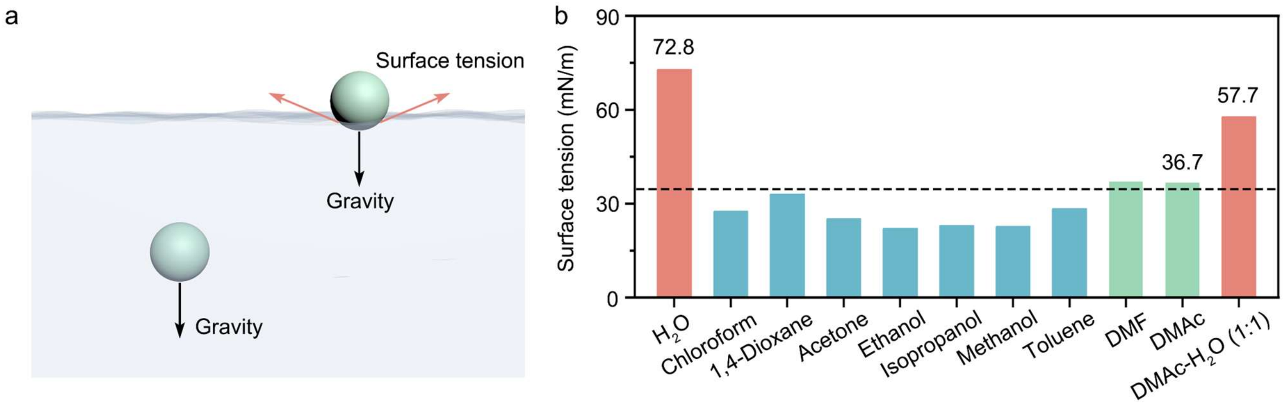

Supplementary Fig. 1| Significance of surface tension for on-liquid surface synthesis. a, Schematic illustration of the liquid surface and its surface tension. **b**, Surface tensions of water, commonly-used organic solvents, and their mixture.

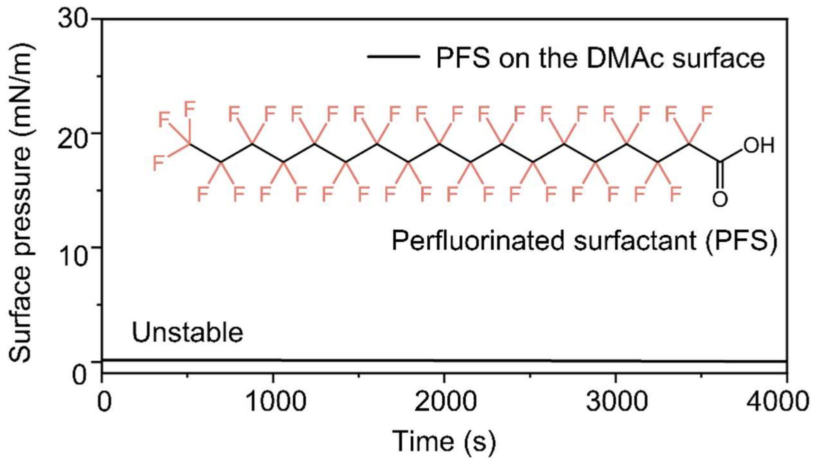

Supplementary Fig. 2| Surface pressure of the DMAc surface during spreading PFS surfactant in a polytetrafluoroethylene (PTFE) trough. The surface pressure is not observed after adding the PFS, demonstrating the unstable feature of PFS on the DMAc surface.

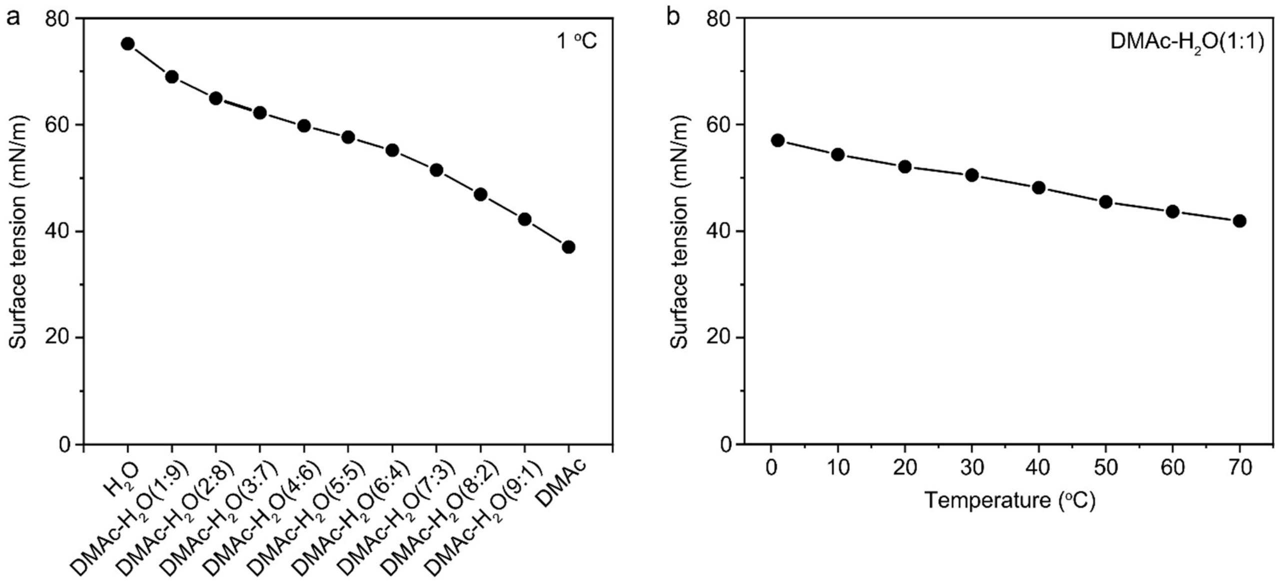

Supplementary Fig. 3| Surface tensions of DMAc-H_2O mixtures. a, Surface tensions of the DMAc-H_2O mixture with different volume ratios at 1 °C. **b**, Surface tensions of DMAc-H_2O mixture (*v*:*v*=1:1) at different temperatures.

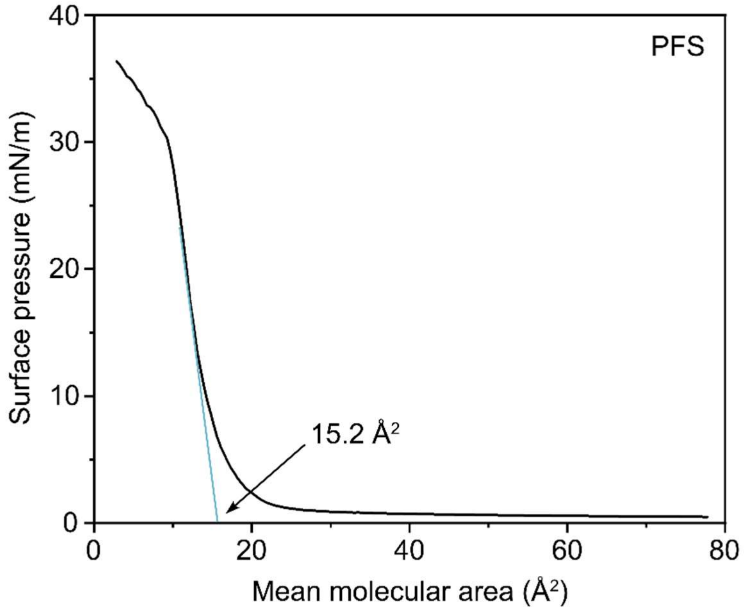

Supplementary Fig. 4| Surface pressure-mean molecular area (MMA) isotherms of PFS on the DMAc-H_2O surface in a Langmuir trough. The MMA of the PFS molecule was calculated to be ~15.2 Å^2.

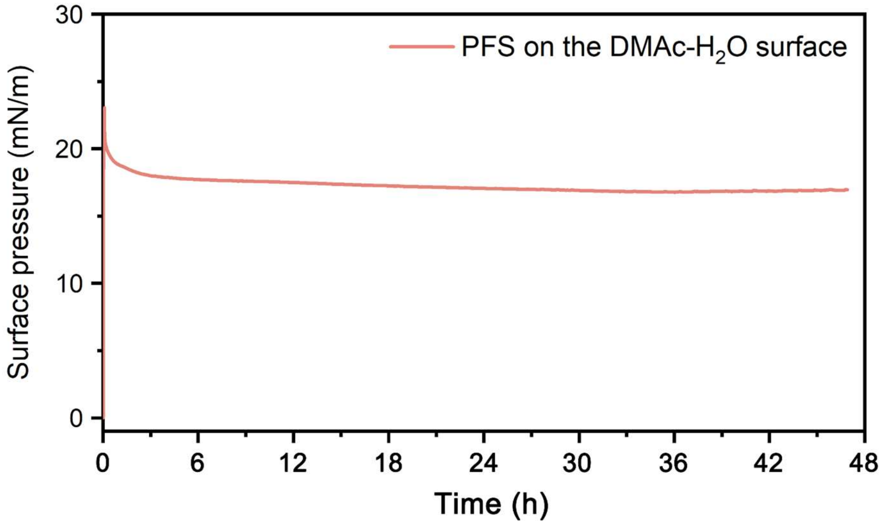

Supplementary Fig. 5| Surface pressures of the PFS monolayer on the DMAc-H_2O surface in a PTFE trough. The surface pressure is constant at ~17 mN m^{-1} even after 2 days, demonstrating the high stability of the PFS monolayer on the DMAc-H_2O surface.

In our study, the formation of stable and crystalline PFS monolayer is critical for driving the accumulation of Cu^+ and oriented crystal growth on the DMAc-H_2O surface. In light of this, we highlight that sufficient surface tension is a prerequisite for supporting amphiphilic PFS surfactants on the DMAc-H_2O surface, facilitating their self-assembly into stable monolayer that can guide the 2D polymerization[10]. In contrast, solvents with low surface tension (e.g., DMAc, $CHCl_3$, EtOH, 1,4-dioxane; typically < 35 mN m^{-1}) cannot stabilize PFS monolayer, leading to its diffusion into the bulk or precipitation (Supplementary Fig. 6).

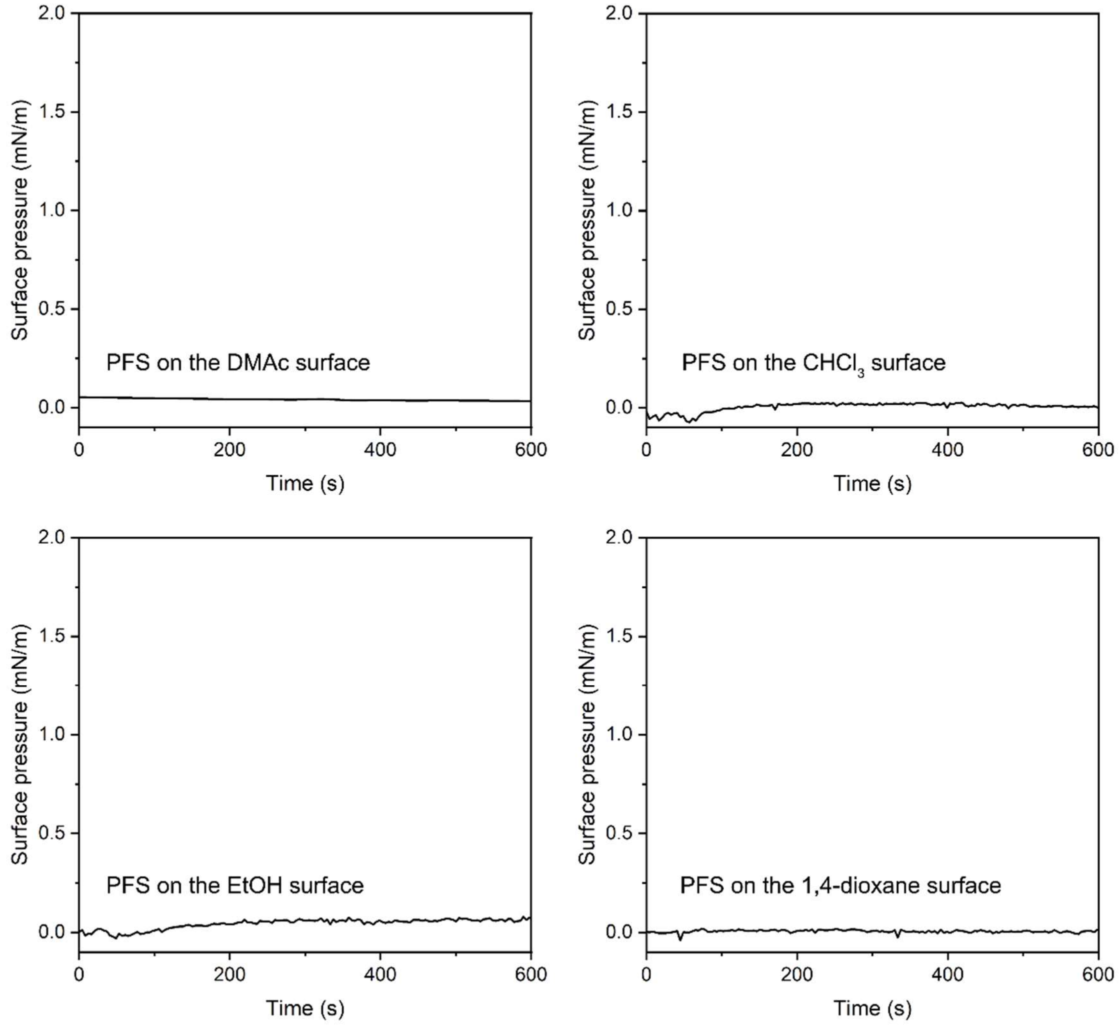

Supplementary Fig. 6| Surface pressures of the DMAc, $CHCl_3$, EtOH and 1,4-dioxane surface after adding PFS molecules.

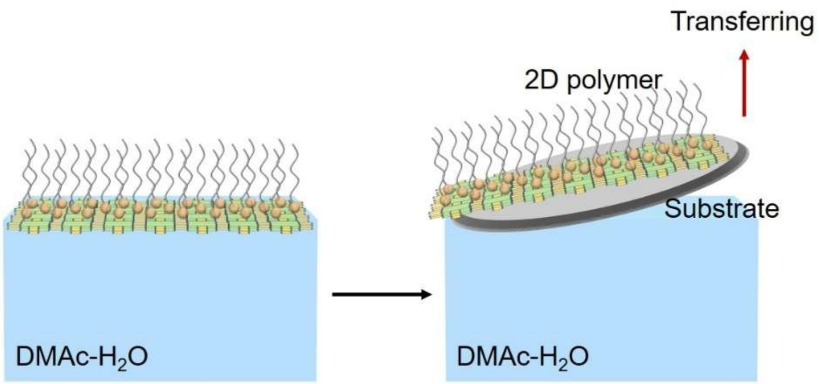

Supplementary Fig. 7| Schematic illustration of the horizontal transfer process.

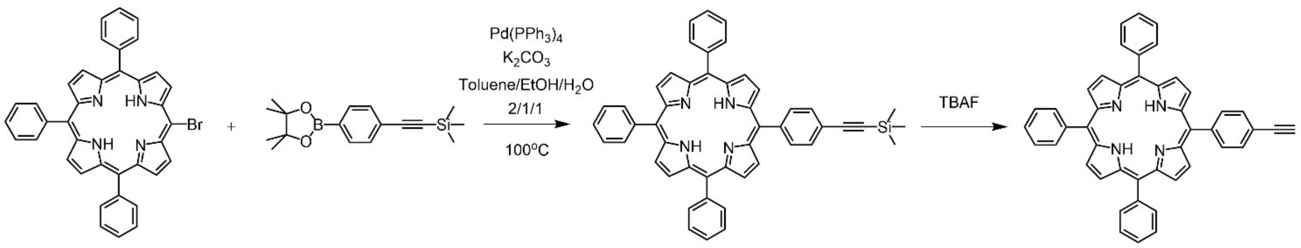

Supplementary Fig. 8| Synthesis of 5,10,15-triphenyl-20-(4-((trimethylsilyl)ethynyl)phenyl)-porphyrin (1). Synthetic route towards **1**.

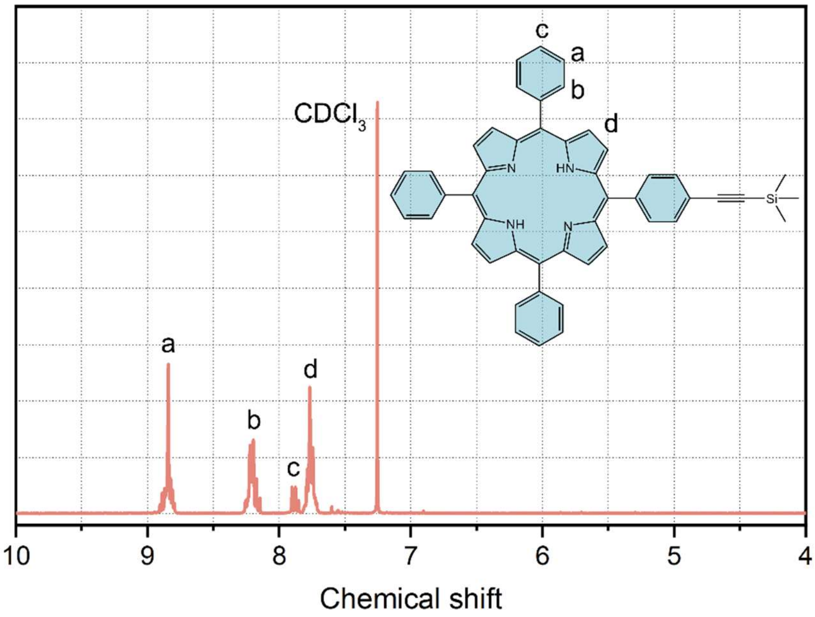

Supplementary Fig. 9| ^{1}H-NMR spectrum of the resultant 5,10,15-triphenyl-20-(4-((trimethylsilyl)ethynyl)phenyl)porphyrin. The compound was dissolved in $CDCl_3$ for the NMR measurement.

The formation of an organic thin film was observed on the DMAc-H_2O surface after the model reaction. To differentiate between bulk and on-liquid surface reactivity, we employed two separate sampling strategies. To obtain the products on the DMAc-H_2O surface, the films were transferred onto the SiO_2/Si substrate via a horizontal dipping approach, followed by vacuum drying at 80 °C (Supplementary Figs. 10b,c). To isolate the bulk-phase products, 2 ml of the subphase liquid was extracted from the bottom of the crystallization dish using a syringe and dried under vacuum at 80 °C, yielding a solid residue deposited on the inner wall of the vial (Supplementary Figs. 10a,b). The collected materials were subsequently re-dissolved in $CHCl_3$ and $CDCl_3$ for matrix-assisted laser desorption/ionization-time-of-flight mass spectrometry (MALDI-TOF MS) and nuclear magnetic resonance (NMR) measurements, respectively.

To further support the surface product formation is not a result of hydrophobic accumulation of products from the subphase, we performed the identical model reaction in the absence of PFS monolayer on the DMAc-H_2O surface. After 24 hours of reactions, we conducted on-liquid surface sampling and attempted to collect any compounds formed on the DMAc-H_2O surface. As shown in Supplementary Fig. 11, no detectable product was observed on the substrate, indicating that no product formed in the subphase and subsequently migrated to the liquid surface via hydrophobic effects. These findings verify that the reactivity on the DMAc-H_2O surface is decoupled from that in the bulk solution, and that the observed product by MS arises only from the on-liquid surface reaction.

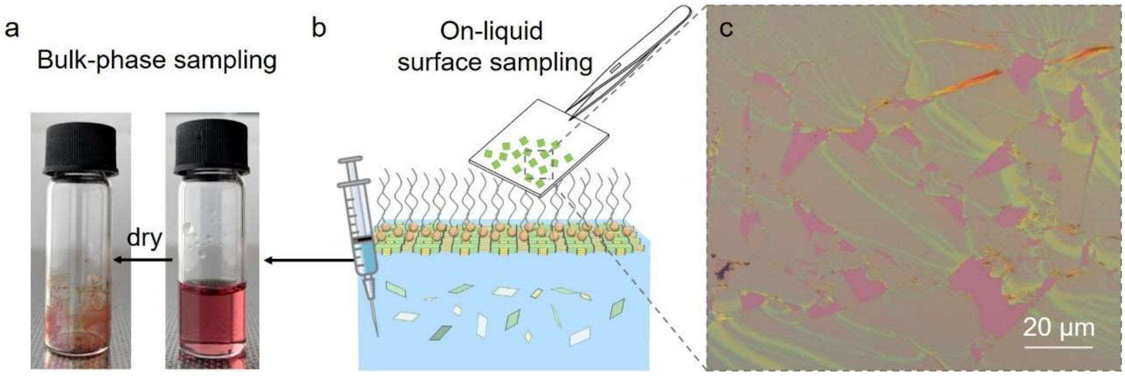

Supplementary Fig. 10| Bulk and on-liquid surface sampling strategies. a, Photographs of the extracted subphase liquid from the bottom of the crystallization dish before and after vacuum drying. **b**, Schematic illustration of on-liquid surface sampling and bulk-phase sampling. **c**, OM image of the Model I product transferred from the DMAc-H_2O surface onto a SiO_2/Si substrate.

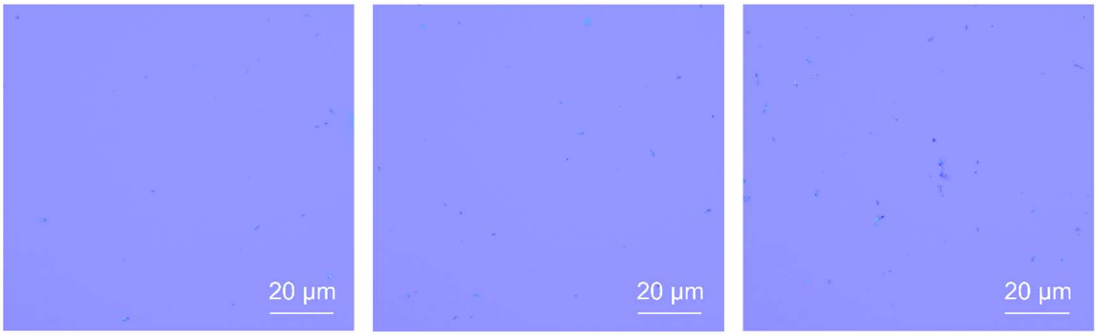

Supplementary Fig. 11| OM images indicate no product formed on the DMAc-H_2O surface in the absence of PFS monolayer.

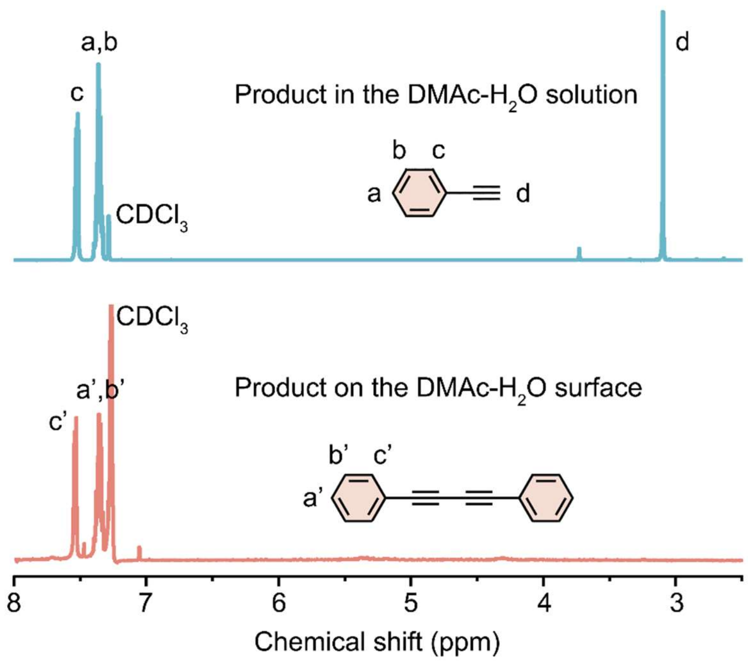

Supplementary Fig. 12| ^{1}H-NMR spectra of the model reaction products on the DMAc-H_2O surface and in bulk solutions. The disappearance of the peak for terminal alkynes demonstrates the higher reactivity of the Glaser coupling reaction on the DMAc-H_2O surface compared to that in its solution phase.

The Glaser coupling reaction undergoes a typical ‘oxidative coupling mechanism’[11,12]. Typically, oxygen transiently oxidizes Cu^{+}-acetylide species (**3a**) to form reactive intermediates (**3b**), potentially including Cu^{2+} species (i.e. oxidative coupling mechanisms), which facilitate C-C bond formation (Fig. 2d). Note that the overall catalytic cycle is redox-balanced, in which Cu^{+} ions are regenerated after each coupling event, enabling their continued participation. In addition, nitrogen-donor ligands such as pyridine help stabilize Cu^{+} ions throughout the process, preventing their irreversible oxidation and maintaining catalytic activity.

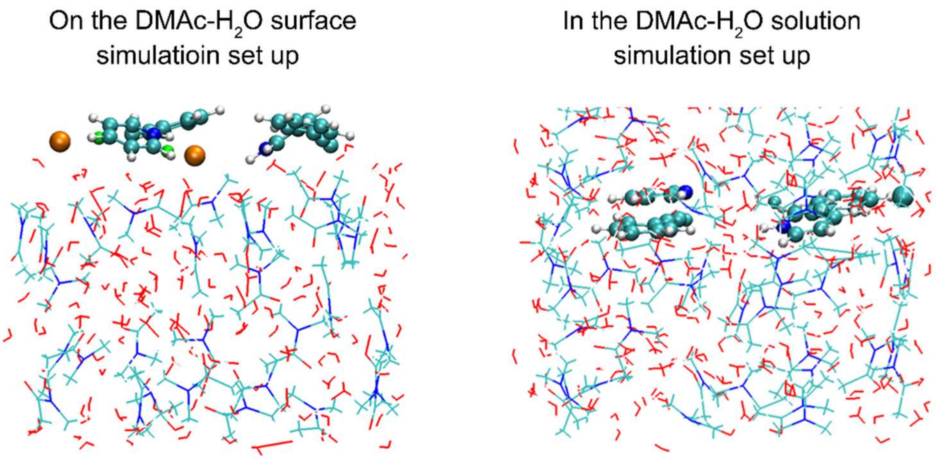

Supplementary Fig. 13| Simulation set up of the Glaser coupling reactions on the DMAc-H_2O surface and in the solution, where the white, teal, blue, red, green, orange colors represent hydrogen, carbon, nitrogen, oxygen, copper, chlorine atoms, respectively. The ratio of DMAc and H_2O molecules is 1:5 in the simulated systems.

The calculated high endothermic barrier of 3.38 eV for the transformation of the Cu^{+}-acetylene intermediate into the diacetylene product underscores the substantial energy barrier associated with this process in the DMAc-H_2O solution. This result is consistent with the MALDI-TOF MS data (Fig. 2b), where only unreacted monomer was detected in the bulk DMAc-H_2O solution, indicating that the formation of the diyne bond is unfavorable.

Importantly, this high energy difference does not imply that the diyne bond would have spontaneously broken in the DMAc-H_2O solution, as the calculation was based solely on two representative intermediate states to estimate the associated energy barriers. Additional intermediate states, such as the dicopper^{2+}-diacetylide complex with various molecular conformations or even the dicopper^{3+}-diacetylide intermediate, likely exist between the diacetylene-linked dimer and Cu^{+}-acetylene complex, and these may involve higher activation energy barriers[13].

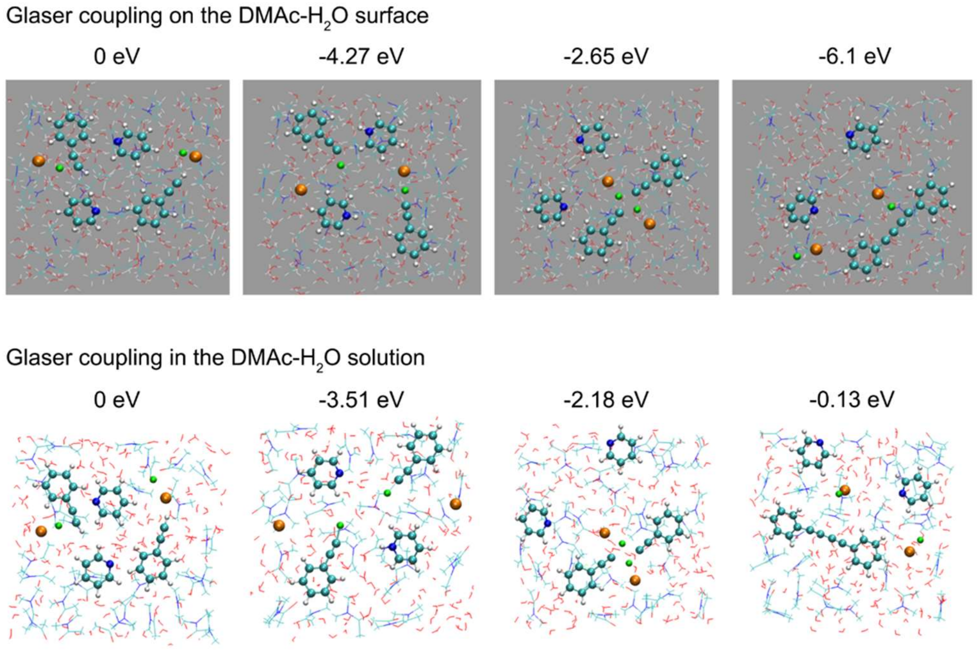

Supplementary Fig. 14| Molecular conformations of the intermediates and their energy barriers. The DFT calculation of the Glaser coupling reaction on the DMAc-H_2O surface and in the solution.

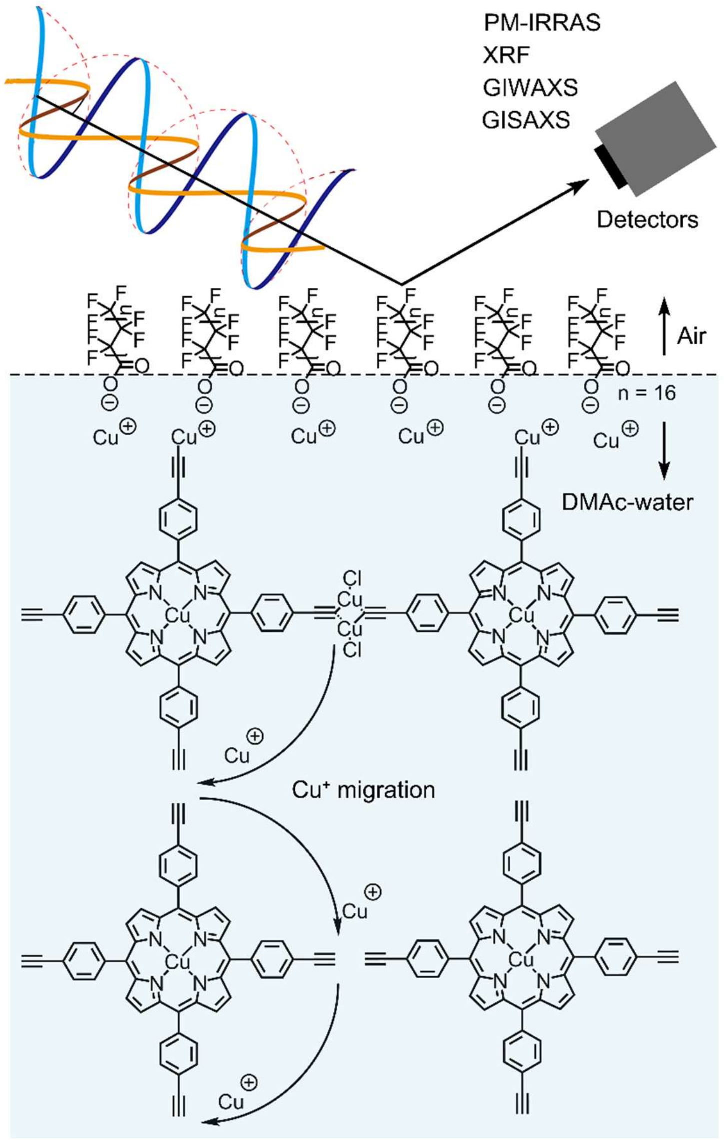

Supplementary Fig. 15| Schematic illustration of in situ IRRAS measurement of 2D polymerization on the DMAc-H_2O surface. The PFS molecules were assembled on the DMAc-H_2O surface to achieve a negative-charged surface. Cu^+ ions as the catalyst for Glaser coupling were adsorbed onto the surface through the electrostatic interaction. The obtained catalyst-rich DMAc-H_2O surface first promotes the 2D polymerization of the TEPP on the surface. Then, the Cu^+ ions migrate downward after catalyzing the top layer, enabling the vertical growth of DY2DPs.

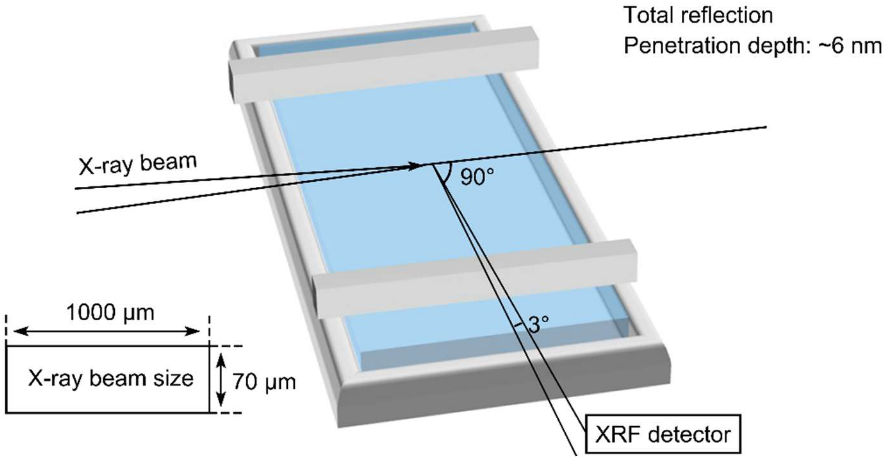

Supplementary Fig. 16| Schematic illustration of the in-situ XRF measurement.

Regarding the new absorption peak at 3,264 cm^{-1}, we note that it emerges exclusively following the addition of the TEPP monomer. Its position, associated with its attenuation during the 2D polymerization strongly suggest assignment to the terminal alkyne C≡C-H stretching. While terminal alkynes typically produce a sharp, well-defined peak in solid-state FTIR (as also observed in our ex-situ FTIR measurement, Supplementary Fig. 33b), the peak on the DMAc-H_2O surface is broadened. This broadening likely arises from polar solvent interactions and heterogeneous local environments around the terminal alkyne groups, which perturb the vibrational dynamics, leading to noticeable band broadening[14,15].

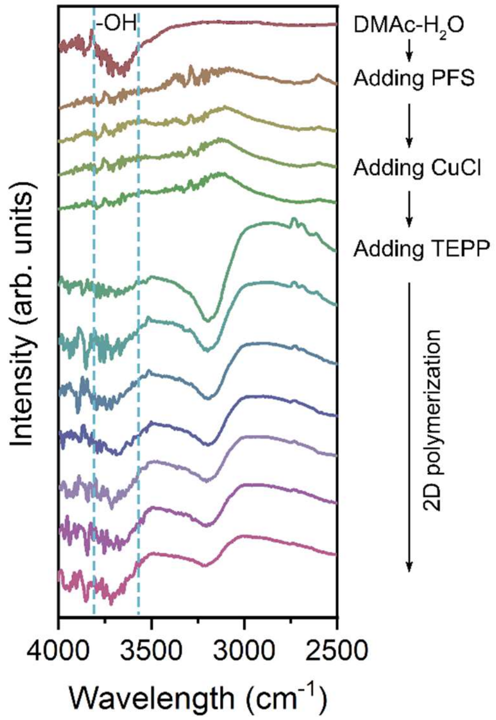

Supplementary Fig. 17| Time evolution of the PM-IRRAS spectra with an angle of incidence α_i = 60° in p-polarization recorded on the DMAc-H_2O surface from Step I to Step III.

We quantified the concentration of Cu^+ ions on the DMAc-H_2O surface using a calibration curve established via in-situ XRF spectroscopy. To construct the calibration, a series of CuCl standard solutions in the DMAc-H_2O mixture with concentrations of 0, 1, 10, and 40 mM were prepared. Then, 50 ml of the prepared CuCl solutions were added into a PTFE trough with a size of 14 cm × 11 cm, respectively, and the corresponding XRF intensities were recorded under identical measurement conditions. The calibration curve of in-situ XRF for Cu^+ ions was obtained as shown in Supplementary Fig. 18. The linear characteristic across various concentrations with a slope of ~838.22 ± 8.83 mM^{-1} was observed. This calibration enabled us to determine the concentrations of Cu^+ ions on the DMAc-H_2O surface from Step I to Step III by converting the recorded XRF signals into absolute concentrations.

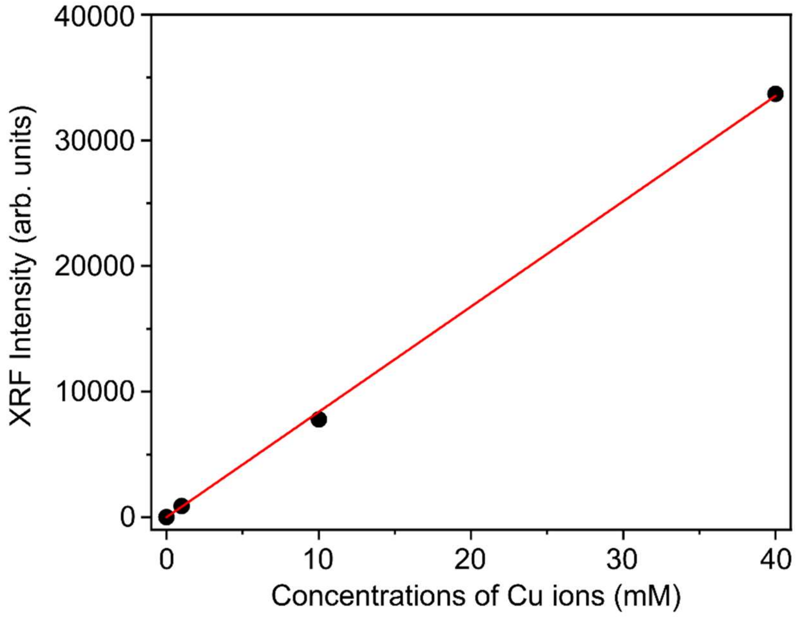

Supplementary Fig. 18| Standard curve of Cu ion solutions measured by in situ XRF.

We have conducted control experiments in the absence of the PFS monolayer and in the presence of stearic acid (SA) to evaluate their role in the 2D polymerization process on the DMAc-H_2O surface. Without the stable PFS monolayer on the DMAc-H_2O surface, the in situ XRF spectra reveal that the Cu^+ ion concentration increases negligibly from 0 to 1.49 mM in Step II, indicating a homogenous ion diffusion process instead of the accumulation process driven by electrostatic interaction (Supplementary Figs. 19-21). Upon the addition of TEPP monomers, no detectable GIWAXS and GISAXS signals were observed in either the in-plane or out-of-plane directions, indicating the absence of oriented monomer assembly or growth of DY2DP-Por crystals (Supplementary Figs. 29-31). Even after 24 hours, no product was formed on the DMAc-H_2O surface (Supplementary Fig. 27). In the system using SA, we note that it cannot form the stable monolayer on the DMAc-H_2O surface, as confirmed by the isotherm measurements of surface pressure (Fig. 1b). After 24-hour polymerization, we did not observe any product formed on the DMAc-H_2O surface through the OM imaging (Supplementary Fig. 28). These results underscore the crucial role of the PFS monolayer in directing the accumulation of Cu^+ and oriented growth of DY2DP-Por crystals.

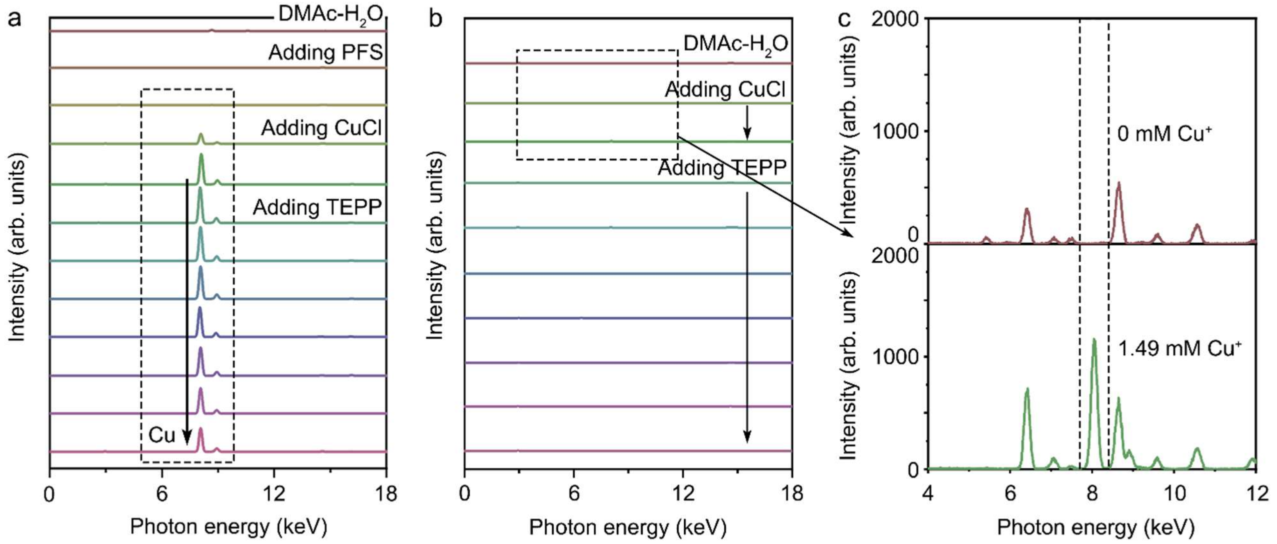

Supplementary Fig. 19| XRF measurement of Cu^+ ion concentrations on the DMAc-H_2O surface with and without PFS monolayer. a,**b**, Time evolution of the XRF spectra recorded on the DMAc-H_2O surface (**a**) with and (**b**) without the PFS monolayer. **c**, In situ XRF spectra of the DMAc-H_2O surface before and after adding CuCl without PFS monolayer. The Cu^+ ion concentration increases negligibly from 0 to 1.49 mM, indicating the homogenous ion diffusion process instead of the adsorption process.

To enable direct comparison of Cu^+ ion adsorption on the DMAc-H_2O surface with and without PFS monolayer, the XRF curves after adding CuCl solution for 4 hours were plotted on a logarithmic scale (Supplementary Fig. 20). The PFS monolayer promotes electrostatically driven adsorption of Cu^+ ions, leading to a Cu^+-rich DMAc-H_2O surface and an ultrahigh intensity of the XRF peak (2.2×10^5) (Fig. 3c). In contrast, without PFS monolayer, Cu^+ ions do not aggregate on the DMAc-H_2O surface, resulting in a much weaker XRF intensity (1.3×10^3), which appears almost as a flat line in Supplementary Fig. 19b.

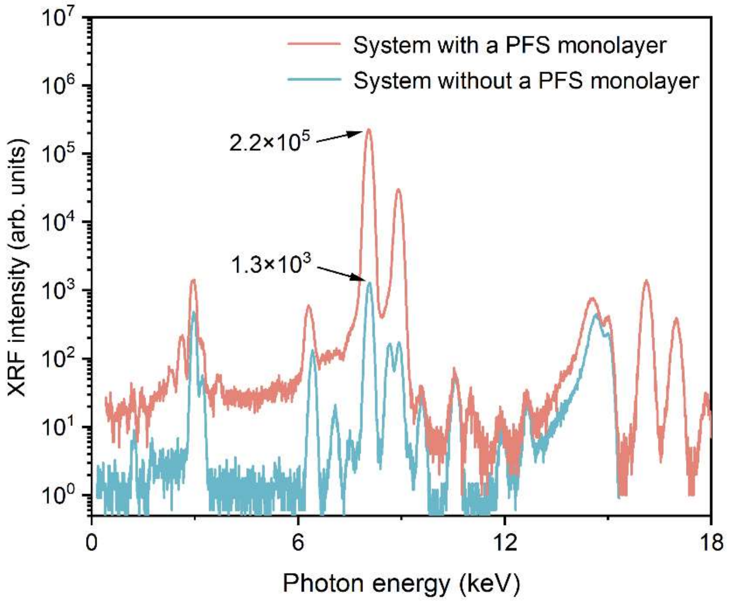

Supplementary Fig. 20| XRF spectra of the reaction system with and without PFS monolayer after adding CuCl solution for 4 hours on a logarithmic scale.

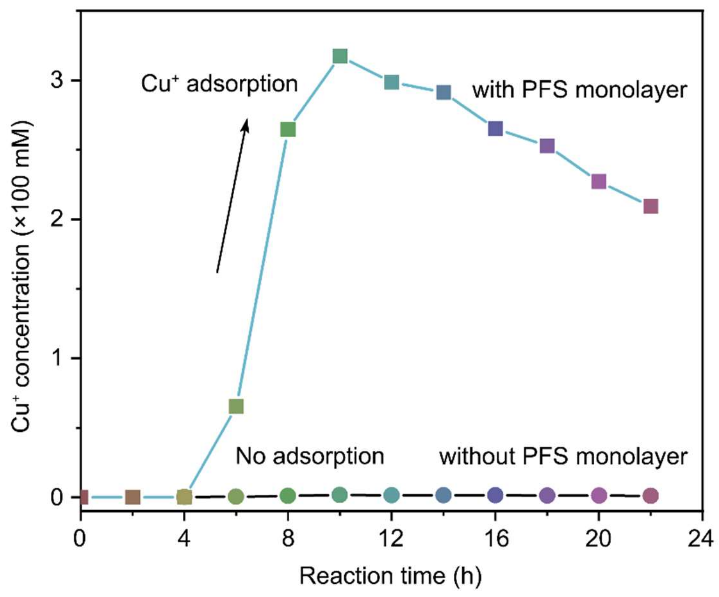

Supplementary Fig. 21| Time evolution of the Cu^+ ion concentration on the DMAc-H_2O surface with and without PFS monolayer. The PFS-assembled DMAc-H_2O surface enables the ultrafast adsorption of Cu^+ ions compared to that without PFS monolayer.

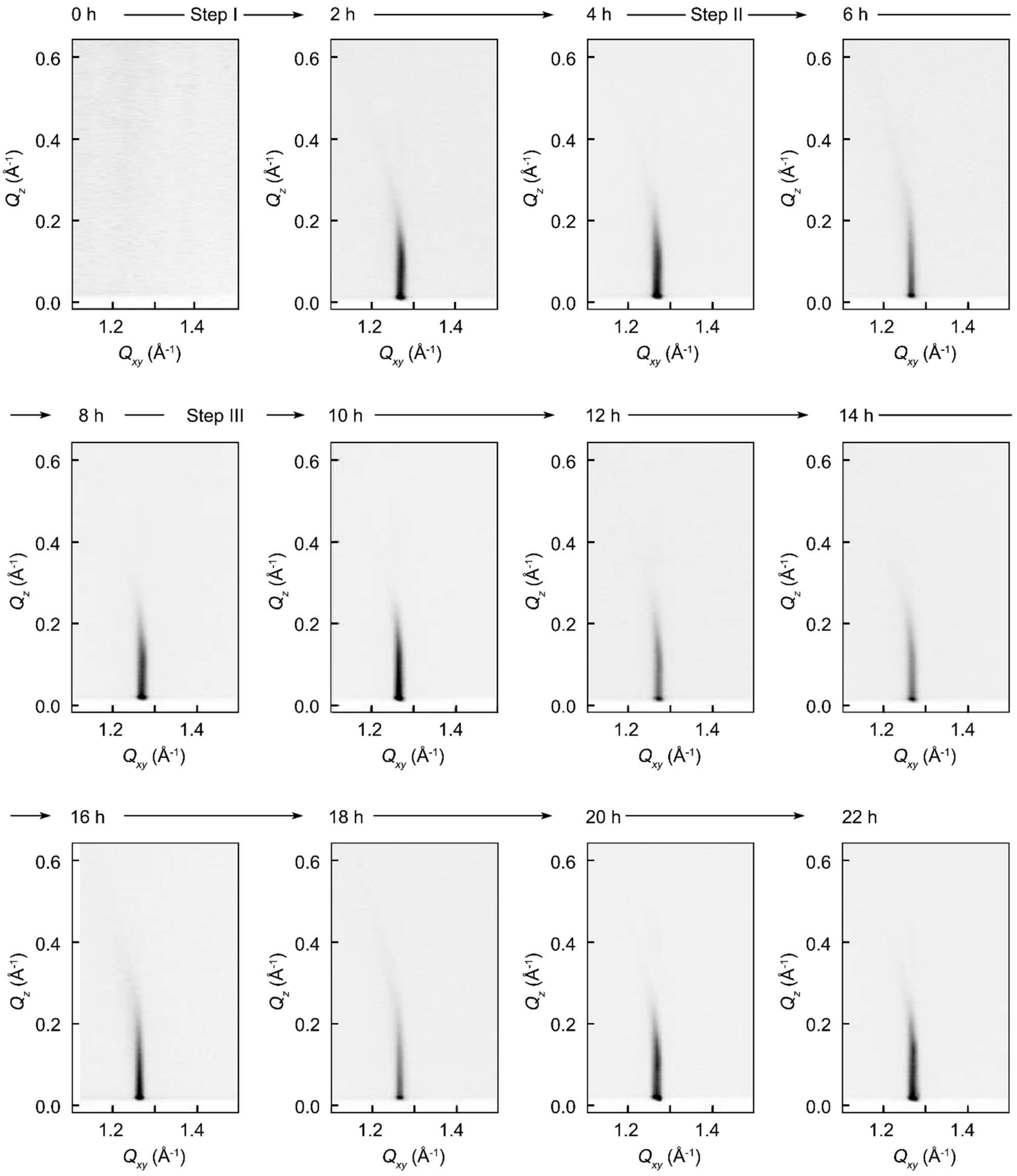

Supplementary Fig. 22| Time evolution of the in situ GIWAXS patterns recorded on the DMAc-H_2O surface from Step I to Step III during the synthesis of DY2DP-Por.

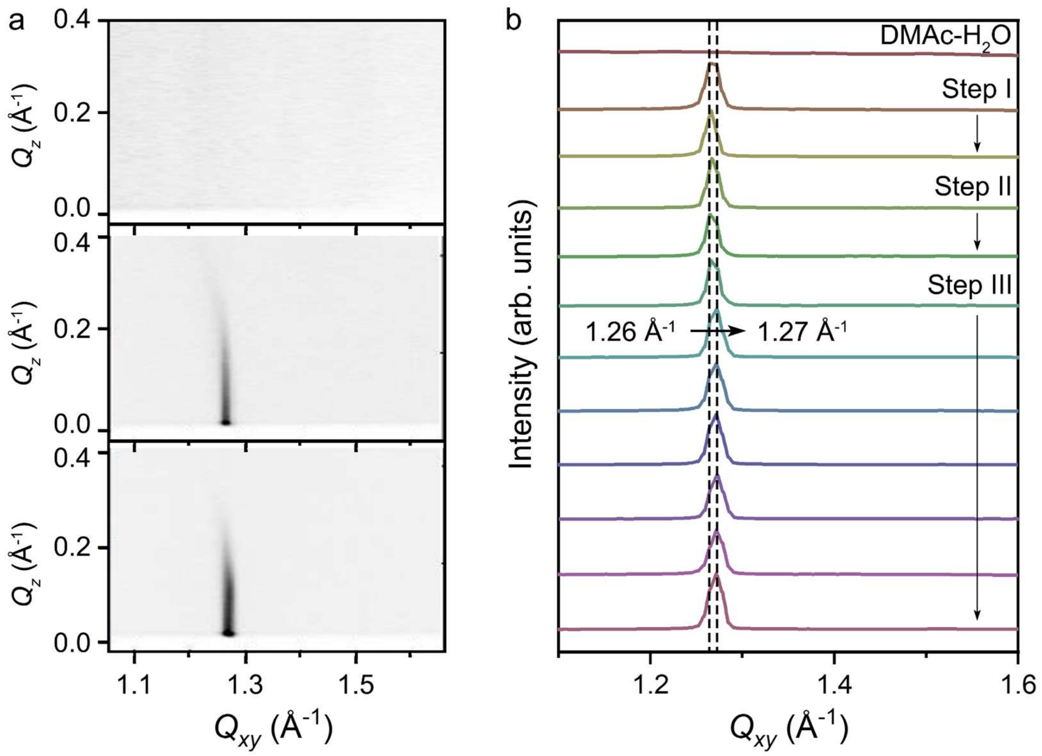

Supplementary Fig. 23| In situ GIWAXS measurement of the 2D polymerization on the DMAc-H_2O surface. a, In situ GIWAXS patterns recorded on the DMAc-H_2O surface before spreading PFS, after spreading PFS and after adding the Cu^+ ions. **b**, Time evolution of the in-plane GIWAXS projections (near $Q_z = 0$, Q represents scattering vector) during Step I-III.

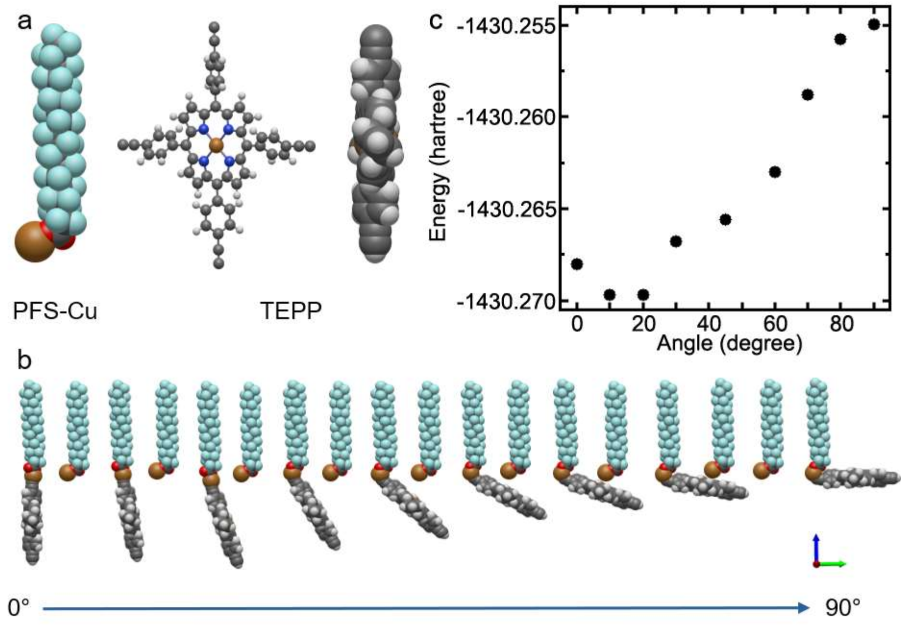

Supplementary Fig. 24| DFT calculations of TEPP molecules underneath a PFS monolayer with different angles. a, Chemical structure of PFS-Cu and TEPP molecules. **b**,**c**, Schematic (**b**) and their energy (**c**) of TEPP molecules underneath a PFS monolayer with different angles.

The observed decrease in the C-F vibrational peak intensity (Fig. 3b) likely results from a combination of dipole reorientation[16] and vibrational mode interference[17]. On the one hand, in Step III, the accumulated Cu^+ underneath PFS monolayer guides the adsorption and assembly of TEPP monomers on the DMAc-H_2O surface, a process that might slightly perturb the local packing environment of the fluorocarbon chains in PFS molecules. This conformational change results in a reduced intensity of the characteristic C-F vibrational peak relative to that observed in Step II, even though the number of bonds remains unchanged. On the other hand, the underlying assembly of TEPP monomers—and the subsequent formation of the 2D polymer crystals—contribute additional vibrational signals. The emergence of these new interfacial species, with distinct dipole orientations and dynamic molecular environments, would further diminish the relative intensity of the C-F band by interfering with its coherent signal generation.

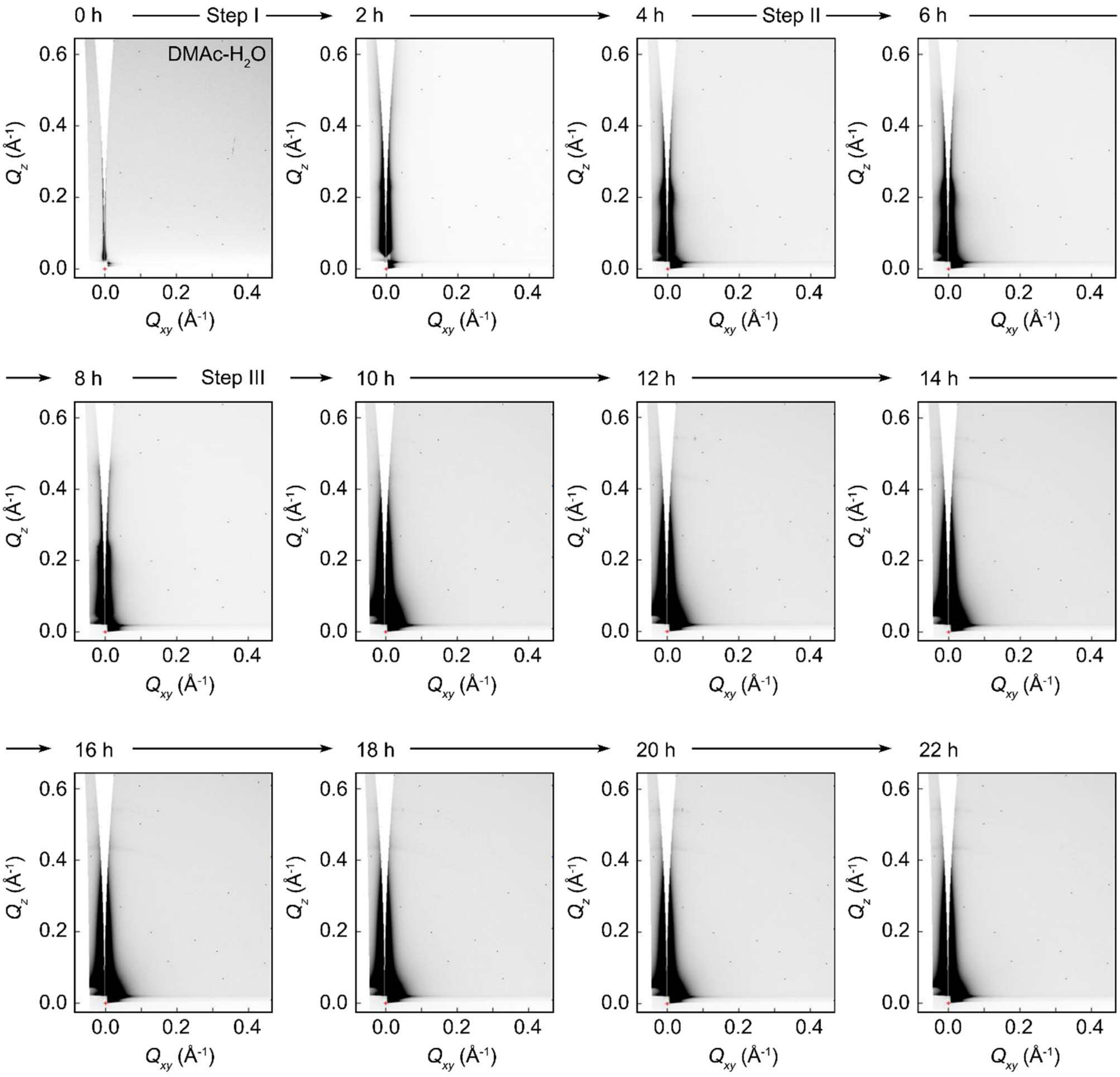

Supplementary Fig. 25| Time evolution of the in situ GISAXS patterns recorded on the DMAc-H_2O surface from Step I to Step III during the synthesis of DY2DP-Por.

In our system, the role of electrostatic interaction is to guide the accumulation of Cu^+ ions underneath the PFS monolayer, forming the Cu^+-rich DMAc-H_2O surface. The spatially confined Cu^+ ions then act as coordination centers that direct the monomer adsorption and assembly via the formation of coordination bonds between Cu^+ ions and terminal acetylene groups, which are known for their directional nature. This coordination-driven assembly can promote crystallographic alignment during polymerization, thereby facilitating epitaxial growth without disrupting the intrinsic intra- and interlayer interactions within the polymer lattice.

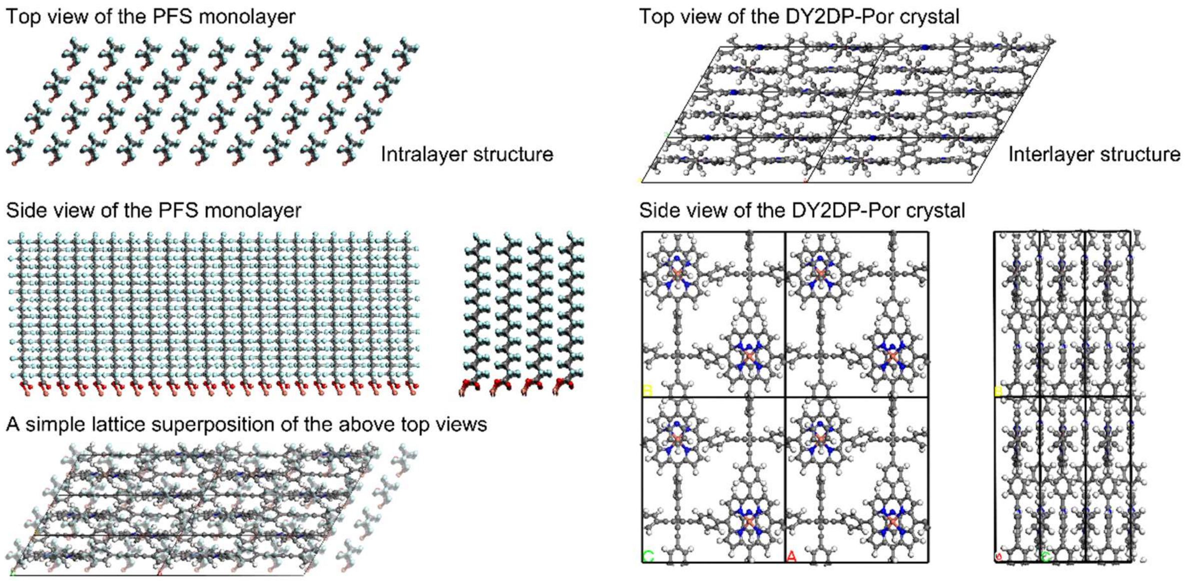

Supplementary Fig. 26| Lattice structures of the PFS monolayer and the DY2DP-Por crystal, and a simple lattice superposition of their top views.

The system without the PFS monolayer

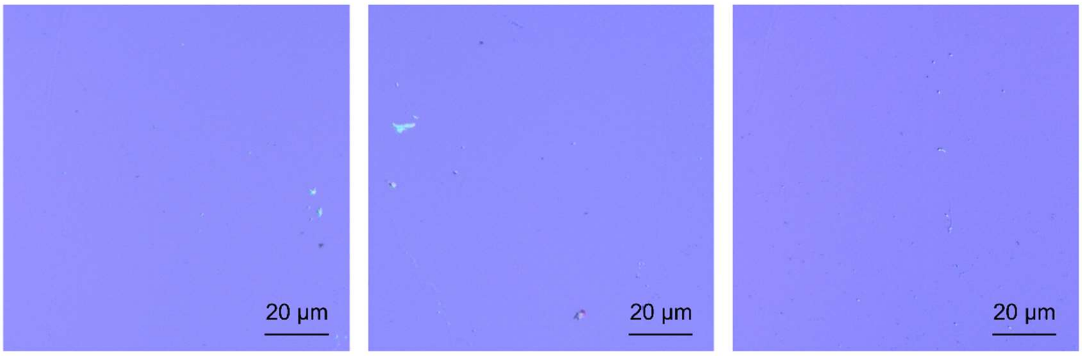

Supplementary Fig. 27| OM images of the products transferred from the DMAc-H_2O surface in the system without the PFS monolayer.

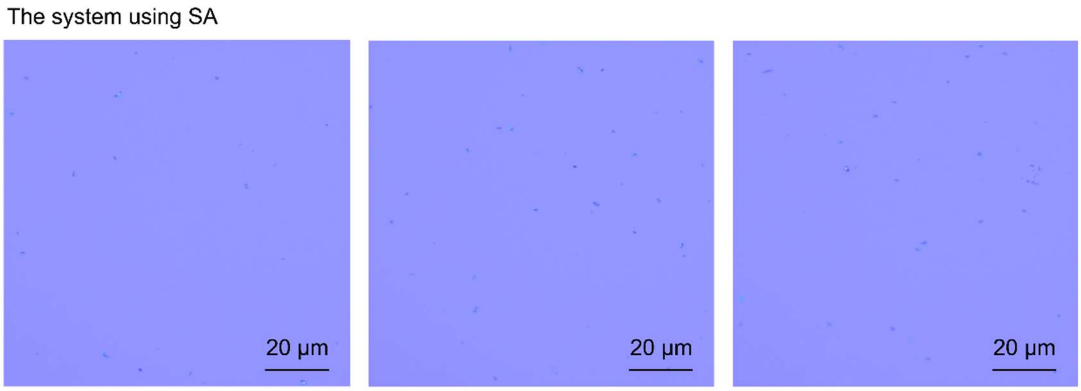

Supplementary Fig. 28| OM images of the products transferred from the DMAc-H_2O surface in the system using SA.

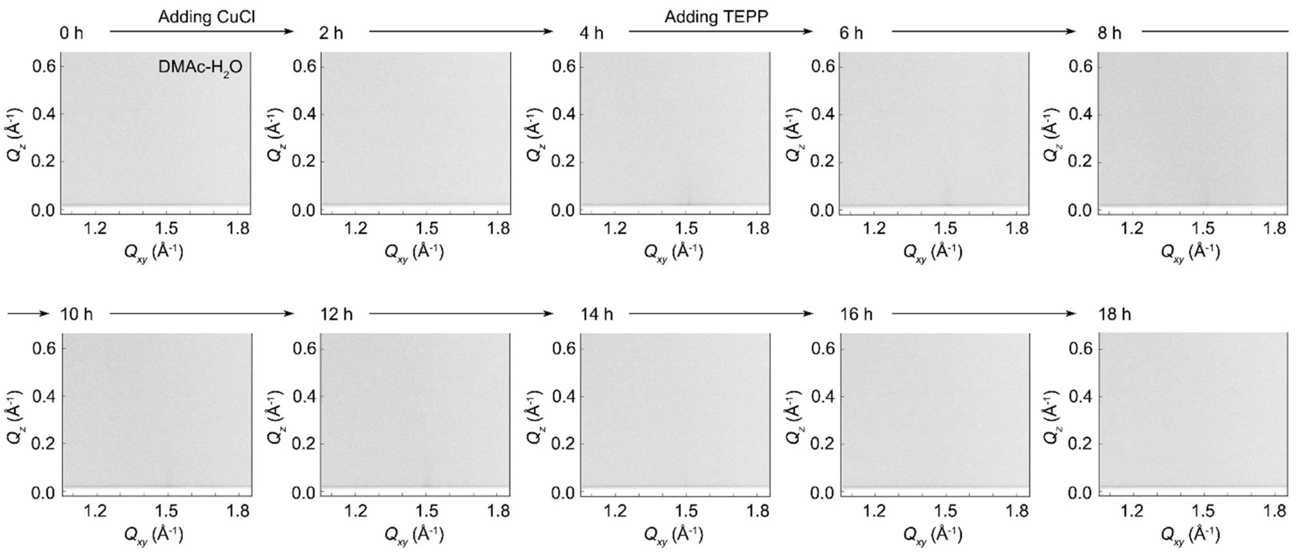

Supplementary Fig. 29| Time evolution of the GIWAXS patterns of 2D polymerization recorded on the DMAc-H_2O surface without PFS monolayer. The weak copper signal was observed 4 hours after adding CuCl, demonstrating the slow diffusion process.

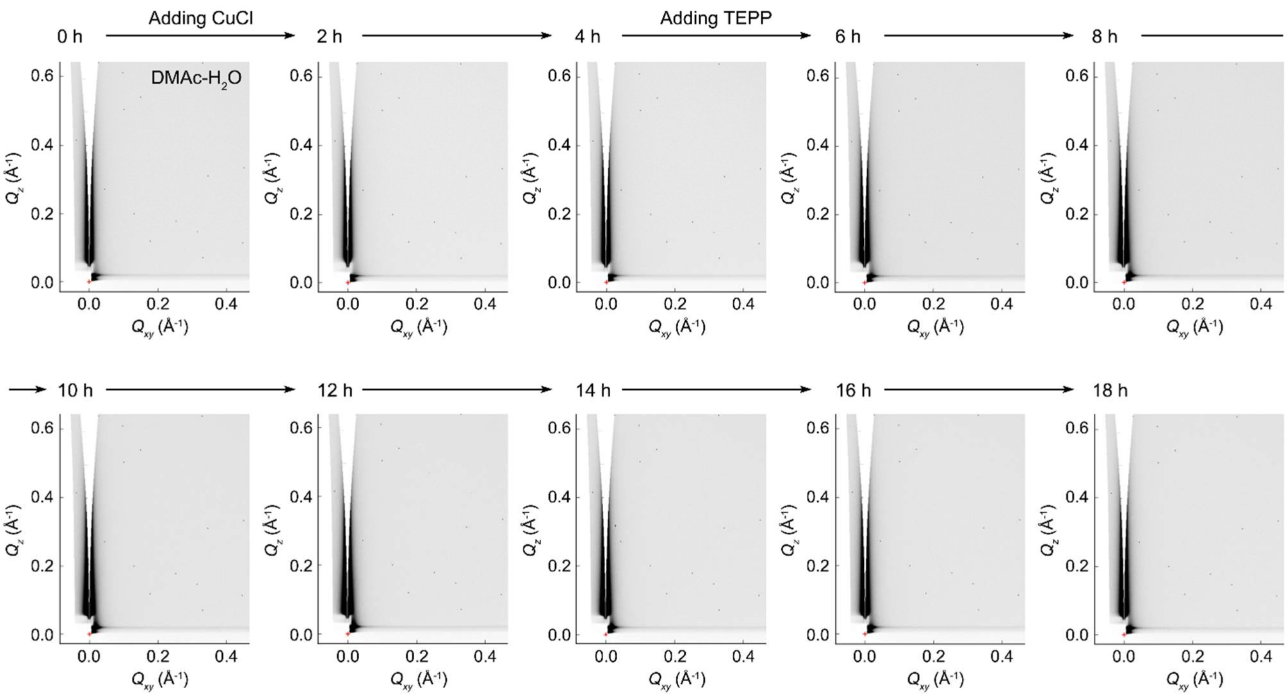

Supplementary Fig. 30| Time evolution of the GISAXS patterns of 2D polymerization recorded on the DMAc-H_2O surface without PFS monolayer.

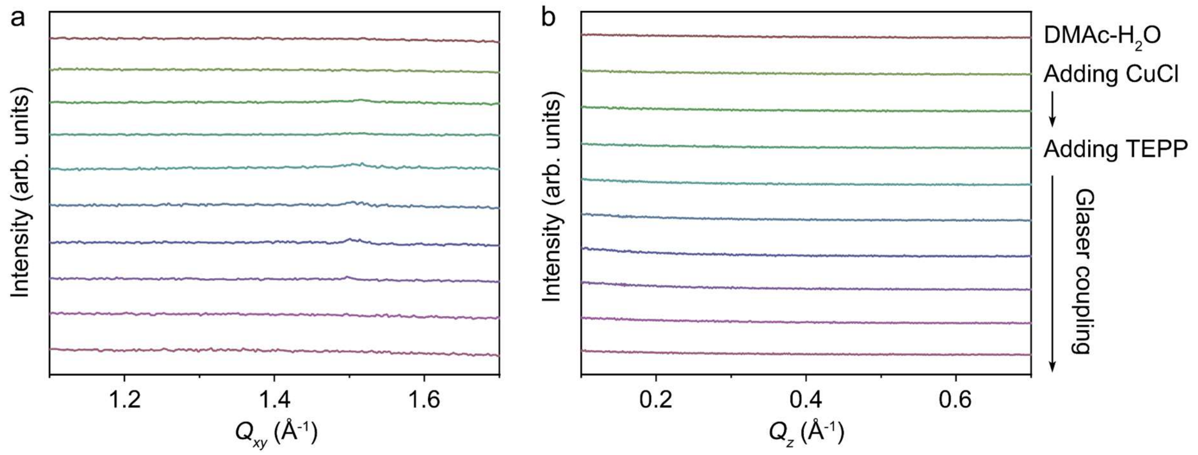

Supplementary Fig. 31| GIWAXS and GISAXS projections of 2D polymerization recorded on the DMAc-H_2O surface without PFS monolayer. a, Time evolution of the in-plane GIWAXS projections (near $Q_z = 0$, Q represents scattering vector) of 2D polymerization recorded on the DMAc-H_2O surface without PFS monolayer. **b**, Time evolution of the out-of-plane GISAXS projections (near $Q_{xy} = 0$) of 2D polymerization recorded on the DMAc-H_2O surface without PFS monolayer.

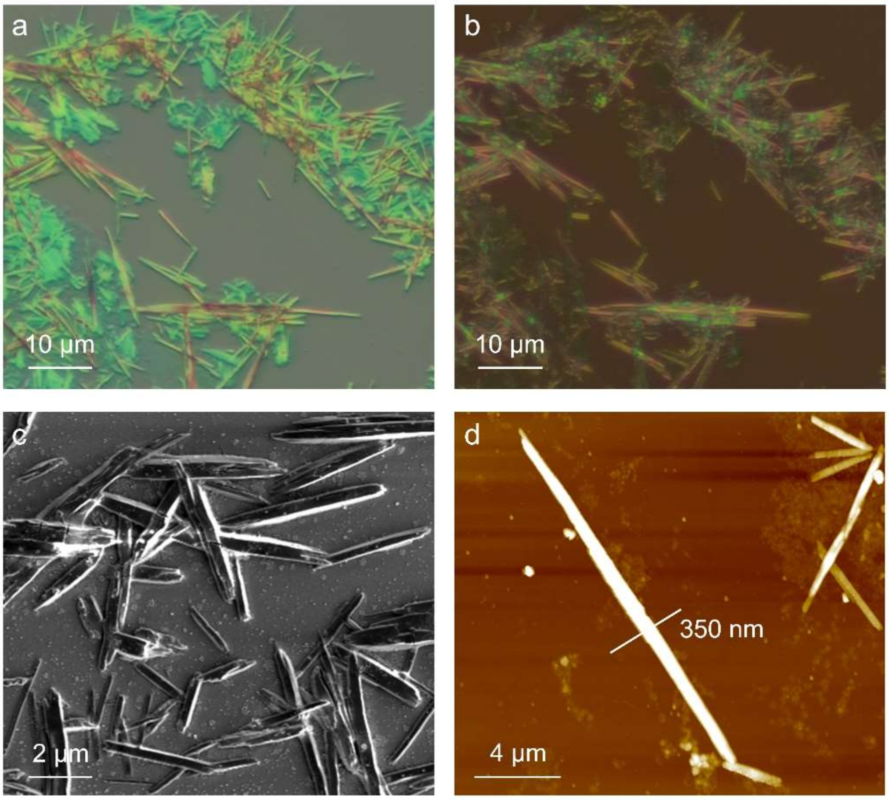

Supplementary Fig. 32| Morphological characterization of DY2DP-Por crystals. a,b,c,d, OM (**a**), polarizing OM (**b**), SEM (**c**) and AFM images (**d**) of rod-shape DY2DP-Por crystals on SiO_2/Si substrates.

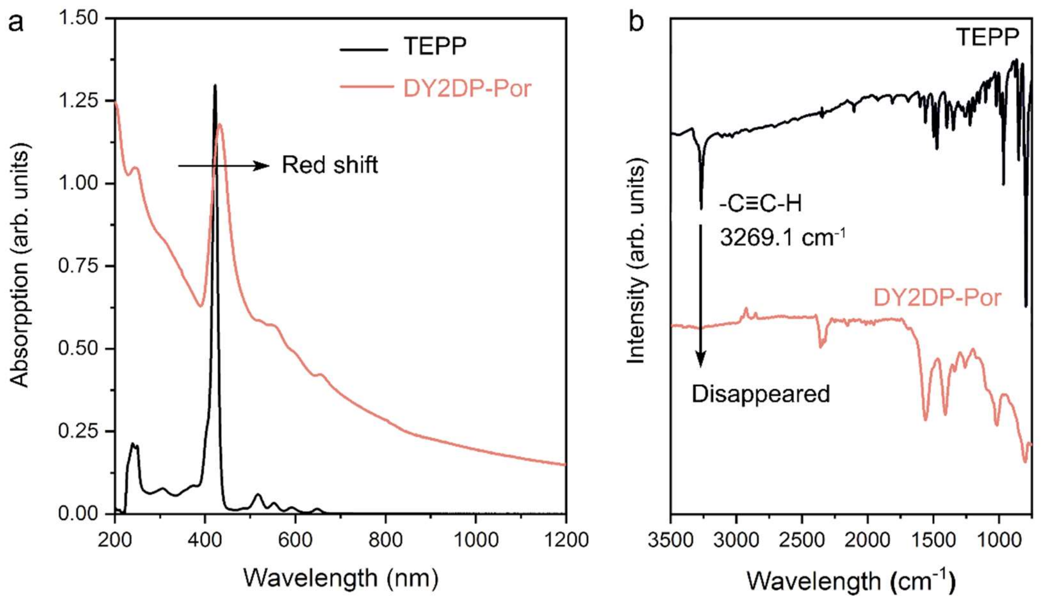

Supplementary Fig. 33| Chemical characterizations of DY2DP-Por. a,**b**, UV-vis (**a**) and ATR-FTIR (**b**) spectra of TEPP (monomer) and DY2DP-Por crystals.

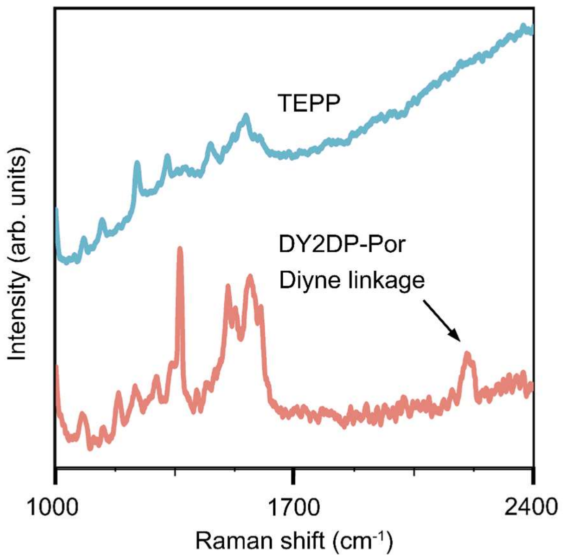

Supplementary Fig. 34| Raman spectra of TEPP (monomer) and DY2DP-Por crystals.

We attribute the observed C=O signal in the XPS spectra to surface-adsorbed carbon dioxide and oxygen molecules, which may interact with the samples surface through either physisorption or chemisorption[18,19]. Considering that the detection depth of XPS is typically limited to only a few nanometers, even an ultrathin layer of adsorbed species or surface oxides can produce a pronounced signal in the measurement.

Regarding the N *1s* spectrum, all the 4 N in the porphyrin macrocycle feature identical peaks. The peaks observed at 401.1 eV, 399.3 eV, and 398.0 eV correspond to the pyridinic N coordinated to Cu (pyridinic N-Cu), N coordinated to Cu in the porphyrin macrocycle ((pyrrolic $N)_4$-Cu), and the pyrrolic N in the uncoordinated H_2-porphyrin macrocycle, respectively. Quantitative analysis reveals that the ratios of (pyrrolic $N)_4$-Cu and pyridinic N-Cu peaks are 7:3, suggesting that the Cu is coordinated at the center of the porphyrin macrocycle with two pyridine molecules as axial ligands, above and below the porphyrin plane. To avoid ambiguity, the corresponding chemical structures have been included in Supplementary Fig. 36b. The XPS survey spectrum of DY2DP-Por (Supplementary Fig. 35) confirms the presence of all expected elements (C, N, Cu).

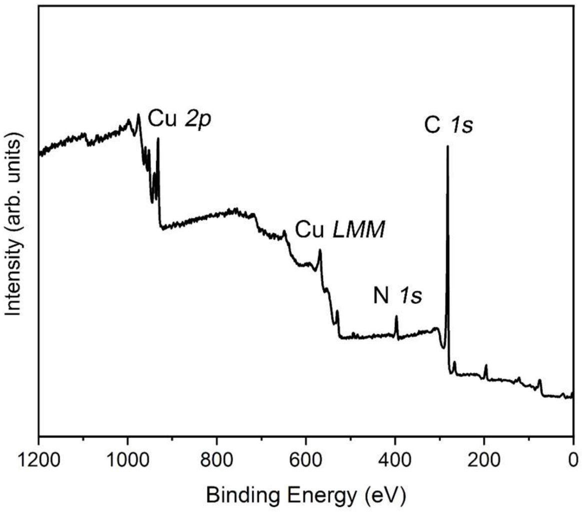

Supplementary Fig. 35| XPS survey scan of DY2DP-Por crystals.

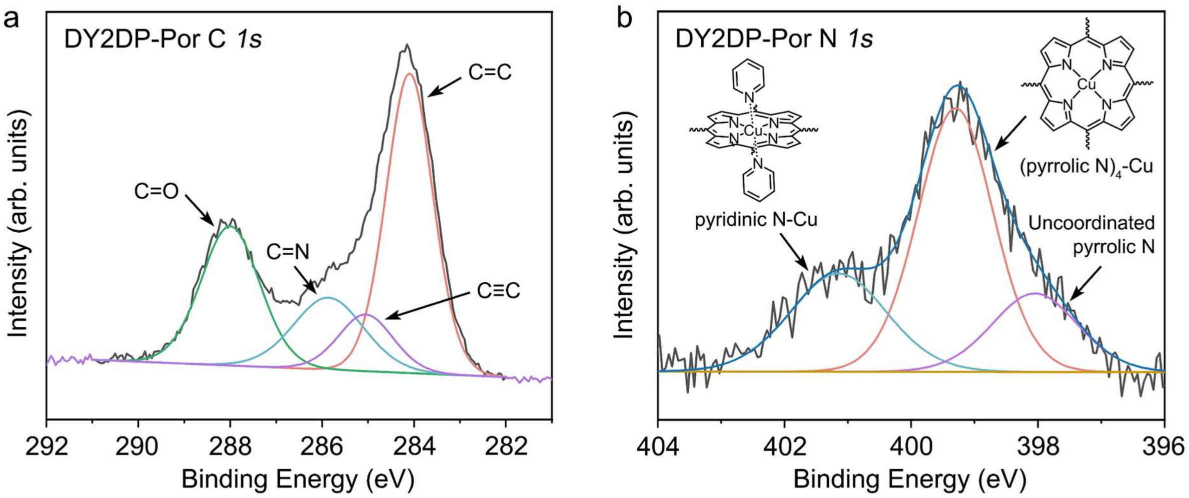

Supplementary Fig. 36| XPS characterization of DY2DP-Por. a,b, Curve-fitted high-resolution XPS C *1s* (**a**) and N *1s* (**b**) spectra of DY2DP-Por crystals.

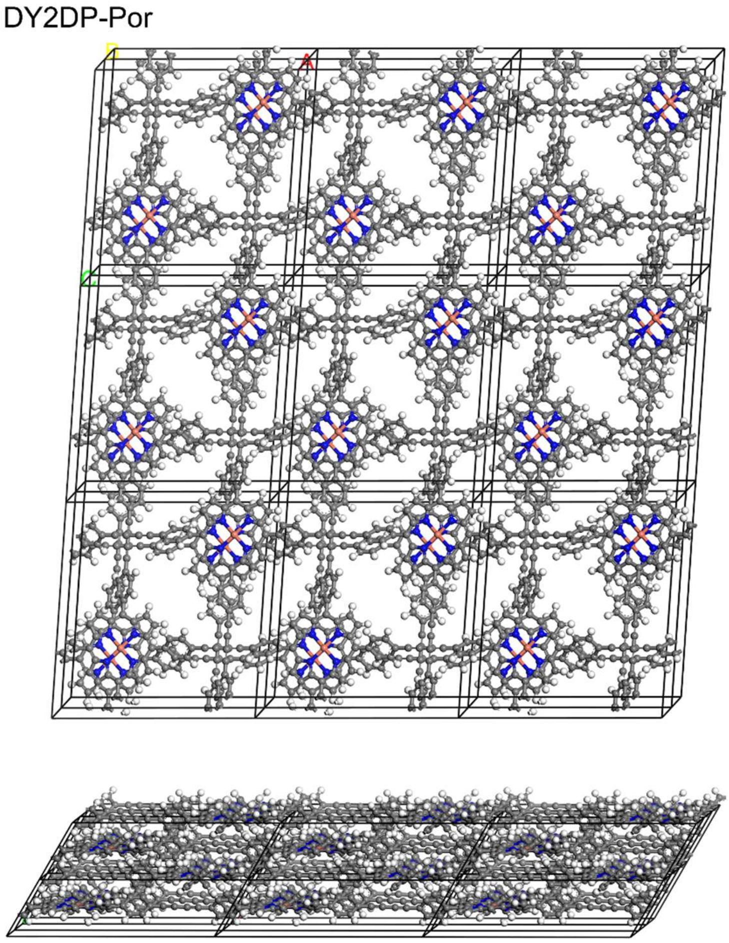

Supplementary Fig. 37| Scheme of the simulated DY2DP-Por structure with inclined AB stacking mode. The crystallographic coordinates are provided in the Source data.

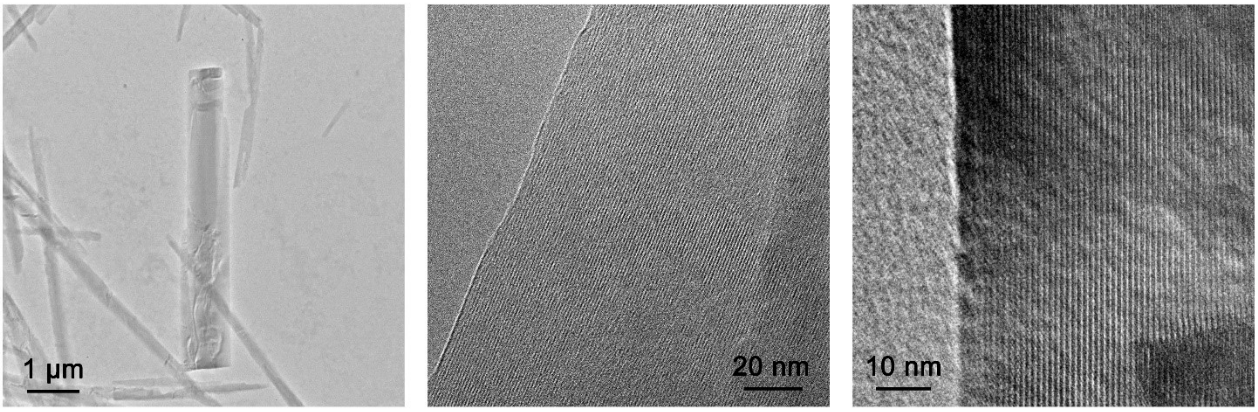

Supplementary Fig. 38| TEM images of DY2DP-Por crystals with different resolutions.

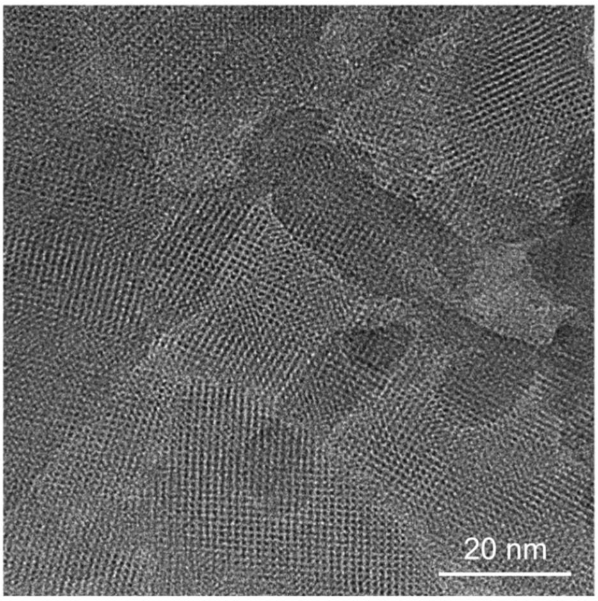

Supplementary Fig. 39| TEM image of the exfoliated DY2DP-Por nanoflakes.

To evaluate the chemical stability, DY2DP-Por crystals were treated with N,N-dimethylformamide (DMF), 1 M HCl and 1 M NaOH aqueous solutions for 7 days. TEM imaging shows that DY2DP-Por retains the integrity of the crystal structure (Supplementary Fig. 40), while Raman spectra reveal that the diyne linkage exhibits a consistent adsorption peak at ~2,202 cm^{-1}, indicating the preservation of the diyne linkage (Supplementary Fig. 41). These results demonstrate that the diyne linkage endows the DY2DP-Por crystals with a robust structure, enabling exceptional chemical resistance even in harsh conditions.

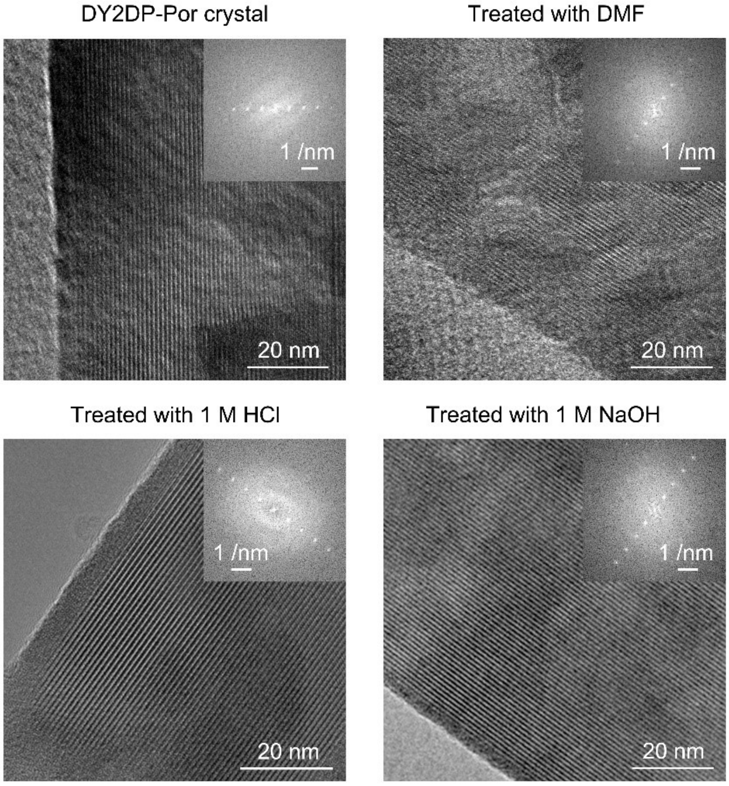

Supplementary Fig. 40| TEM images of DY2DP-Por crystals treated with DMF, 1M HCl and 1 M NaOH. Insets: FFT images of DY2DP-Por crystals treated with DMF, 1M HCl and 1 M NaOH.

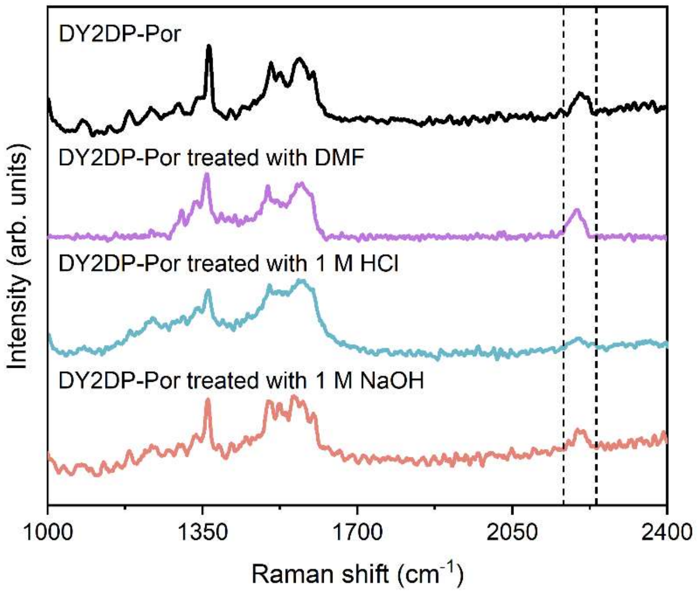

Supplementary Fig. 41| Raman spectra of DY2DP-Por crystals treated with DMF, 1M HCl and 1 M NaOH.

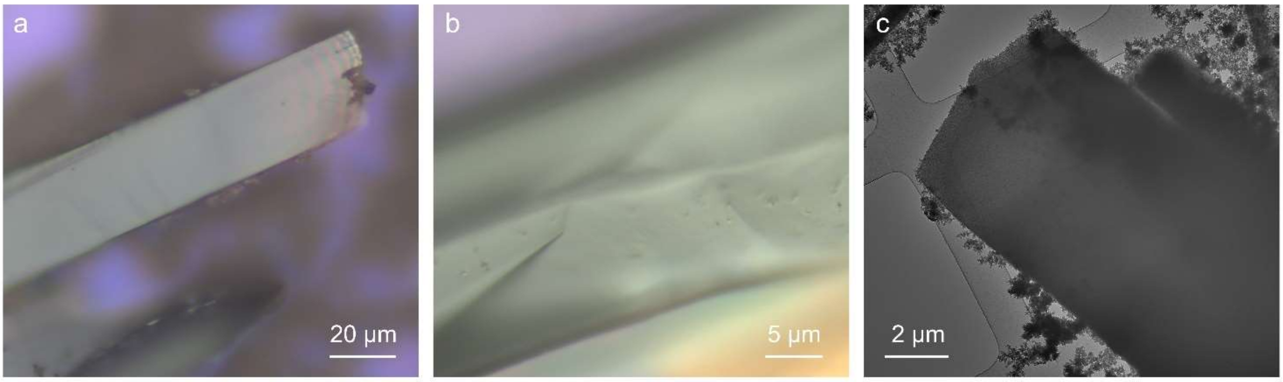

Supplementary Fig. 42| Morphological characterization of GDY crystals. a,**b**, OM images of GDY crystals, showing a rod-shaped morphology. **c**, TEM image of GDY crystals.

The size distributions of DY2DP-Por and GDY crystals have been statistically analyzed. DY2DP-Por crystals exhibit a monomodal size distribution with widths of 0.3-0.7 μm and lengths of 6-14 μm (Supplementary Figs. 43a-c). In contrast, GDY crystals are larger, with widths of 2-9 μm and lengths of 15-50 μm (Supplementary Figs. 43d-f). Both DY2DP-Por and GDY are presented as micrometer-scale rod-shaped crystals.

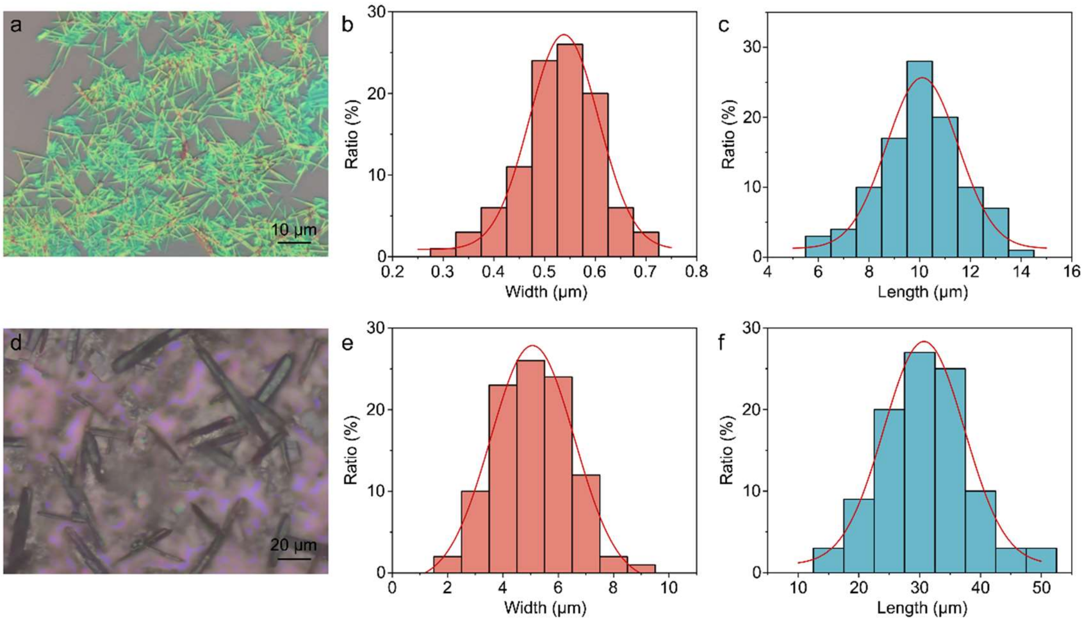

Supplementary Fig. 43| The size distributions of DY2DP-Por and GDY crystals. a, OM image of DY2DP-Por crystals. **b**, Statistical distribution of the width of DY2DP-Por crystals. **c**, Statistical distribution of the length of DY2DP-Por crystals. **d**, OM image of GDY crystals. **e**, Statistical distribution of the width of GDY crystals. **f**, Statistical distribution of the length of GDY crystals.

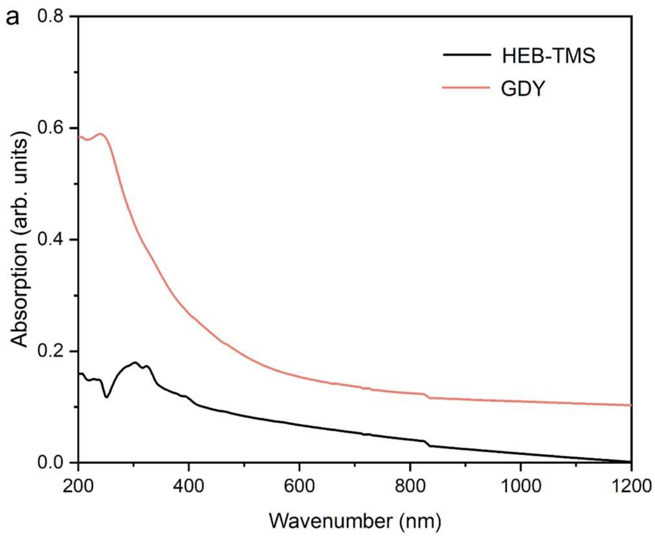

Supplementary Fig. 44| Chemical characterization of GDY. a,**b**, UV-vis (**a**) and ATR-FTIR (**b**) spectra of HEB-TMS (monomer) and GDY crystals.

Under our reaction conditions, the TMS deprotection is facilitated by the presence of excess CuCl, which promotes the formation of Cu^+-acetylene complex and hexamethyldisiloxane[20-22]. To experimentally confirm the removal of TMS group, we further conducted XPS and Raman measurements on the resulting GDY crystals (Supplementary Figs. 45 and 46). The XPS survey spectra shows no detectable silicon signal, demonstrating the efficient deprotection of TMS during the 2D polymerization. Moreover, the Raman peak of HEB-TMS at 2897 cm^{-1}, corresponding to the $-CH_3$ in TMS, disappears completely in the GDY crystals. These results collectively confirm the removal of TMS group during GDY formation.

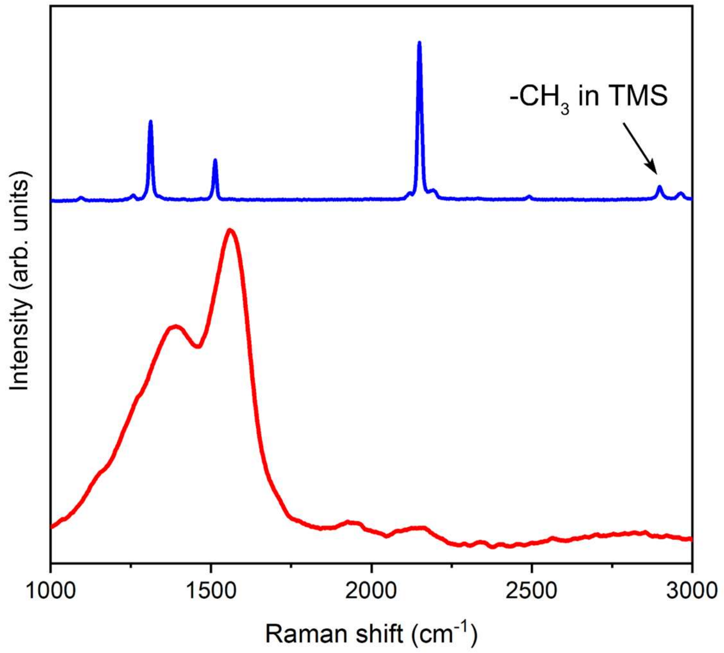

Supplementary Fig. 45| Raman spectra of HEB-TMS (monomer) and GDY crystals.

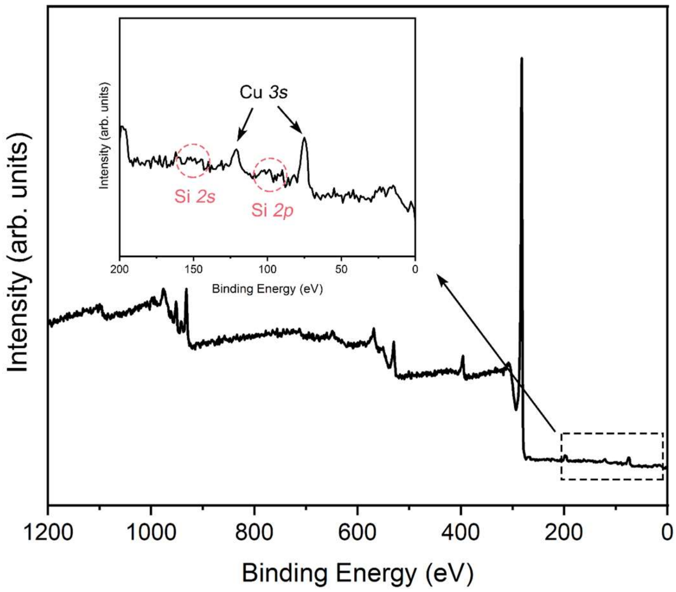

Supplementary Fig. 46| XPS survey scan of GDY crystals. Inset: High-resolution XPS spectra in the binding energy region of 0-200 eV.

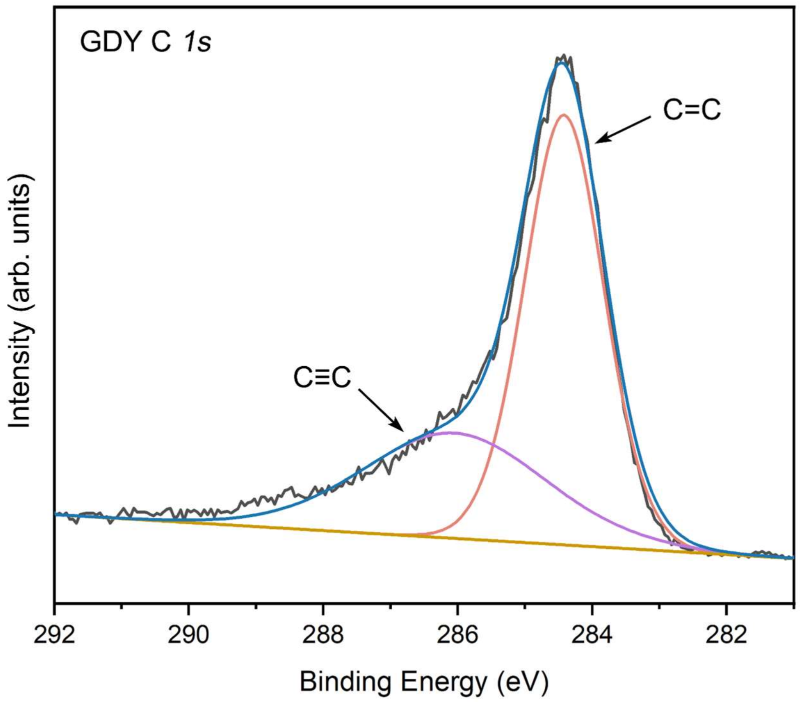

Supplementary Fig. 47| Curve-fitted high-resolution XPS C *1s* spectra of GDY crystals.

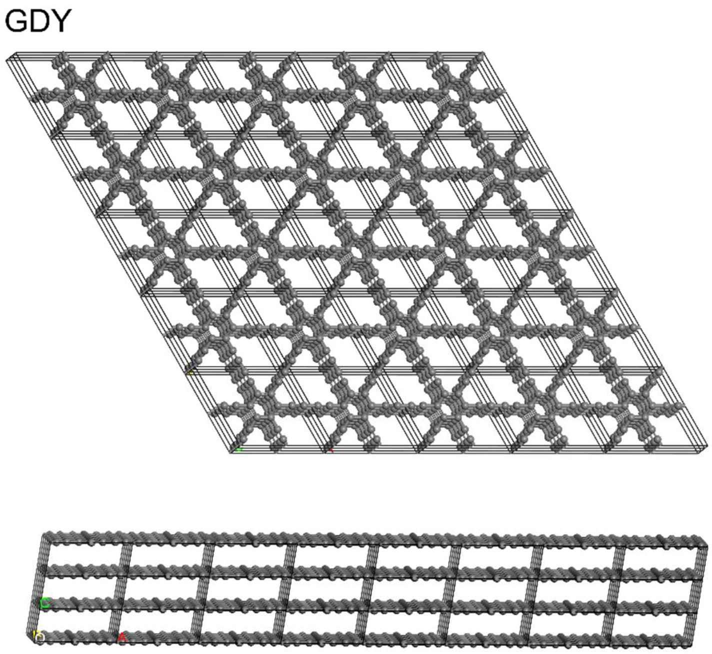

Supplementary Fig. 48| Scheme of the simulated GDY structure with inclined AA stacking mode. The crystallographic coordinates are provided in the Source data.

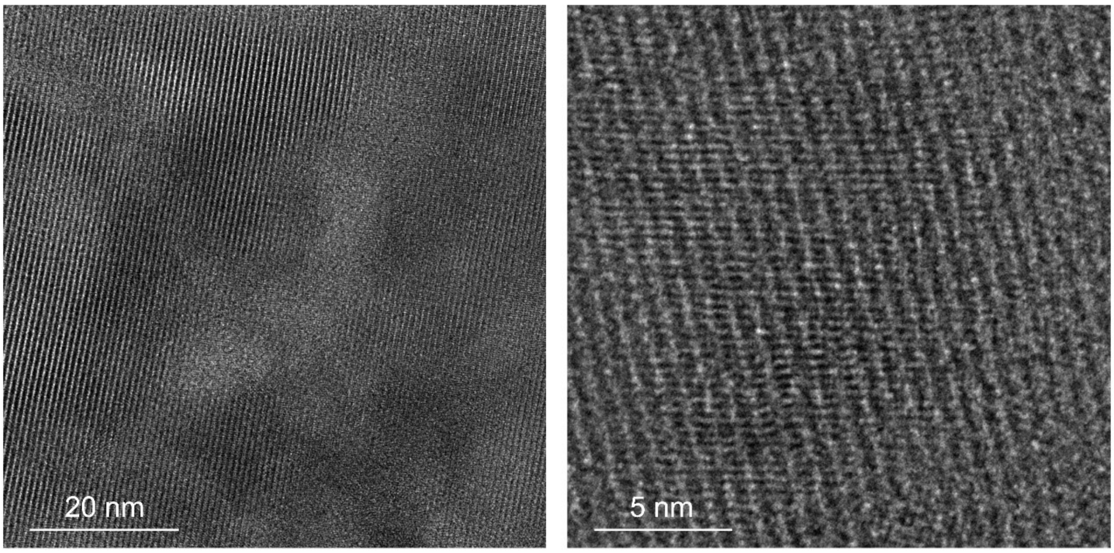

Supplementary Fig. 49| TEM images of GDY crystals with different resolutions.

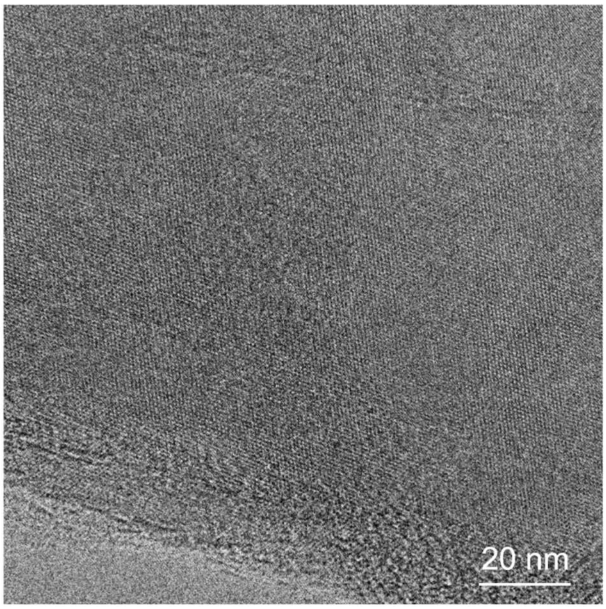

Supplementary Fig. 50| TEM image of the exfoliated GDY nanoflake.

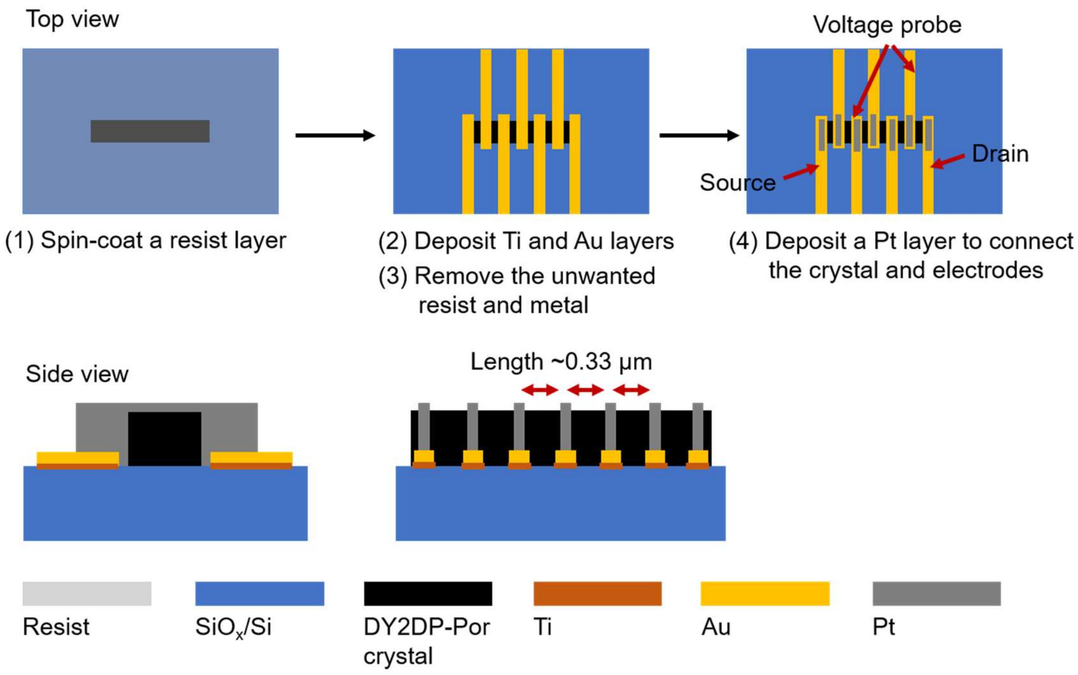

Supplementary Fig. 51| Schematic diagram of DY2DP-Por crystal device fabrication procedure.

The crystallographic direction of DY2DP-Por crystals was determined by TEM and SAED analysis (Supplementary Fig. 52). The SAED pattern reveals diffraction spots corresponding to the (001) plane along the long axis of the DY2DP-Por crystal, indicating that the crystal length aligns with the interlayer stacking direction (Supplementary Fig. 52a). Furthermore, the high-resolution TEM image shows lattice fringes corresponding to the (200) plane along the width of the crystal, confirming that the crystal width corresponds to the intralayer direction (Supplementary Fig. 52b). The corresponding FFT pattern is consistent with the SAED data, further validating the crystal orientation. Note that the electronic measurements were performed along the long axis of the crystal. Thus, the measured conductivity reflects the charge transport along the interlayer direction.

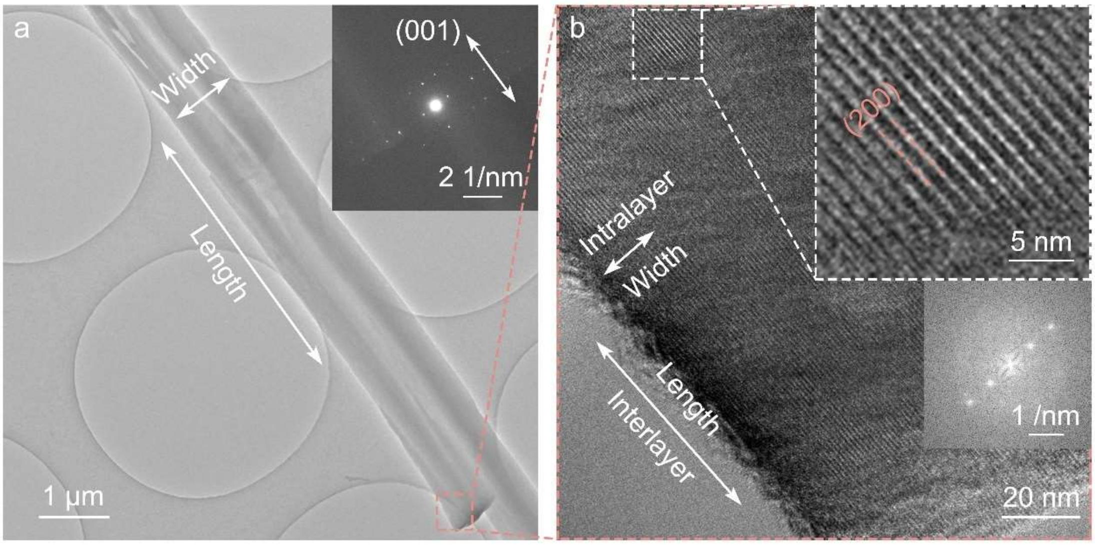

Supplementary Fig. 52| a, TEM image of rod-shaped DY2DP-Por crystals. Inset: SAED pattern of DY2DP-Por crystals. **b, TEM, high-resolution TEM and the FFT images (inset) of rod-shaped DY2DP-Por crystals.**

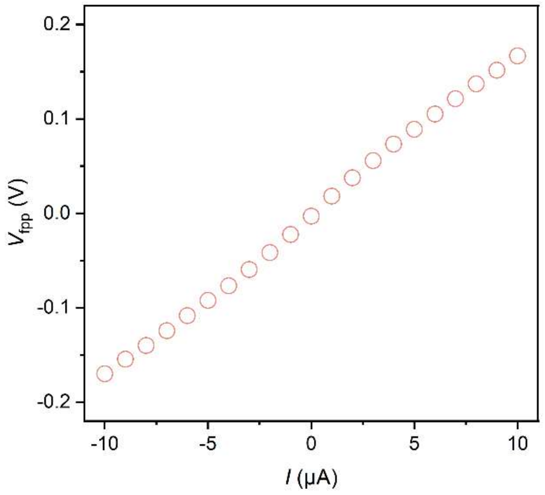

Supplementary Fig. 53| *I-V* curve of the DY2DP-Por crystal device.

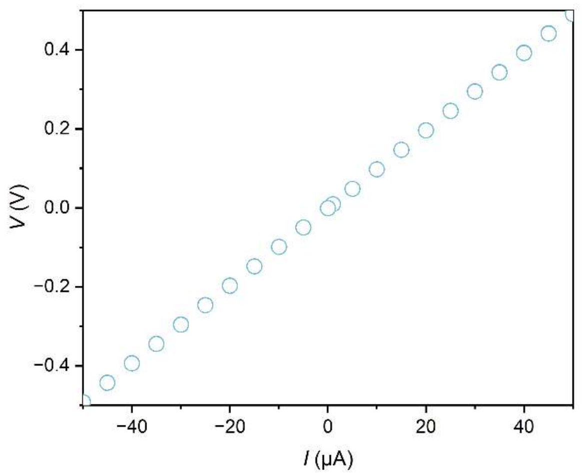

Supplementary Fig. 54| I-V curve of the GDY crystal device.

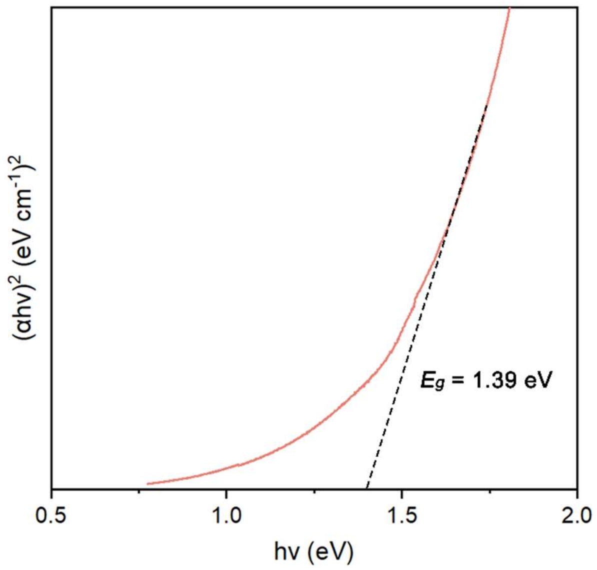

Supplementary Fig. 55| Tauc plot analysis of DY2DP-Por.

To investigate whether the measured conductivity arises from unintentional air doping, we conducted temperature-dependent electron paramagnetic resonance (EPR) on DY2DP-Por at 5, 25, 50 and 100 K (Supplementary Fig. 56). The spectra reveal strong, well-defined signals corresponding to Cu^{2+} ions coordinated within the porphyrin macrocycle of DY2DP-Por. Importantly, no additional EPR features attributable to organic radicals or air-induced dopants were detected across the temperature range studied, thereby ruling out air doping as a contributing factor to the measured electronic conductivity.

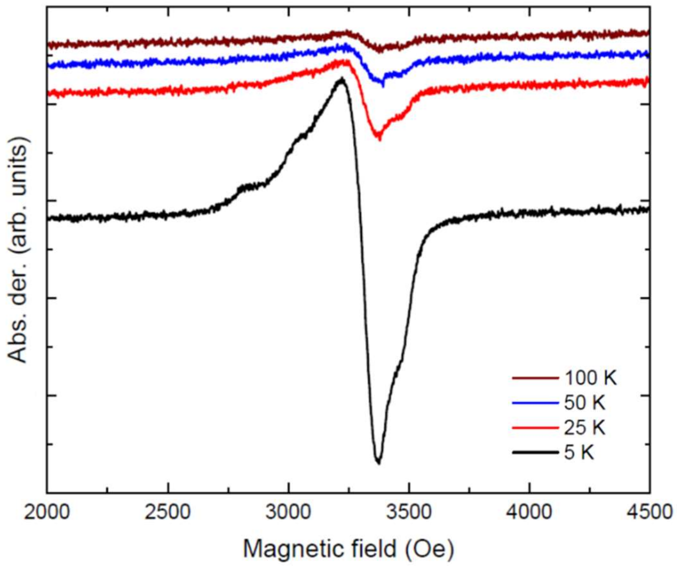

Supplementary Fig. 56| Temperature-dependent EPR spectra of DY2DP-Por at 5, 25, 50 and 100 K using a microwave frequency of 9.56 GHz.

Supplementary Tables

Supplementary Table 1. Summary of the domain size, orientation and stacking mode of the GDY crystals synthesized via the O-SMAIS method and other reported strategies.

Synthetic approaches	Domain size (μm)	Orientation	References
Solvothermal synthesis	~0.002	Random	[23]
CVD method	Amorphous	-	[24]
Explosion method	~0.01	Random	[25]
On-metal surface synthesis	N/A	Face-on	[26]
Liquid-liquid interfacial synthesis	N/A	Face-on	[19]
Gas-liquid interfacial synthesis	~1.5	Face-on	[15]
O-SMAIS	~30	Edge-on	This work

Supplementary Table 2. Summary of the conductivity values of DY2DP-Por, GDY and the literature reported 2D COFs at room temperature.

Material	Type	σ (S cm^{-1})	Ref.
DY2DP-Por	Crystal	0.58	This work
GDY	Crystal	0.36	This work
NiPc-CoTAA	Film	0.0052	[27]
HATN-AQ-COF	Film	0.00022	[28]
PyVg-COF	Film	0.004	[29]
Ni-COF	Pellet	0.013	[30]
Ni-COF	Film	1.2	[30]
TANG-COF	Pellet	0.01	[31]
C2P-5	Film	1.75	[32]
CuPc-AQ-COF	Film	15.3	[33]
PEDOT@AQ-COF	Pellet	1.1	[34]
TFPPy-ICTO-COF	Pellet	0.001	[35]
NiPc-NH-CoPcF8	Pellet	0.0272	[36]
NiPc-NH-CoPcF8	Film	0.127	[36]
TAPFY-COF	Pellet	0.00028	[37]

Supplementary Table 3. Summary of the conductivity values of GDY at room temperature.

Synthetic approaches	Type	Stacking Modes	σ (S cm^{-1})	References
On-metal surface synthesis	Film	ABC	2.52×10^{-6}	[26]
On-metal surface synthesis	Film	N/A	2.42×10^{-8}	[38]
Vapor-liquid-solid synthesis	Nanowire	ABC	19	[39]
CVD method	Film	N/A	6.72	[24]
O-SMAIS	Crystal	AA	0.36	This work

Supplementary References

1 Hutter, J., Iannuzzi, M., Schiffmann, F. & VandeVondele, J. Cp2k: atomistic simulations of condensed matter systems. *Wiley Interdiscip. Rev. Comput. Mol. Sci.* **4**, 15-25 (2014).

2 Perdew, J. P., Burke, K. & Ernzerhof, M. Generalized gradient approximation made simple. *Phys. Rev. Lett.* **77**, 3865 (1996).

3 Goedecker, S., Teter, M. & Hutter, J. Separable dual-space Gaussian pseudopotentials. *Phys. Rev. B* **54**, 1703-1710 (1996).

4 Kühne, T. D. *et al.* Cp2k: an electronic structure and molecular dynamics software package - Quickstep: Efficient and accurate electronic structure calculations. *J. Chem. Phys.* **152** (2020).

5 VandeVondele, J. *et al.* Quickstep: fast and accurate density functional calculations using a mixed Gaussian and plane waves approach. *Comput. Phys. Commun.* **167**, 103-128 (2005).

6 VandeVondele, J. & Hutter, J. Gaussian basis sets for accurate calculations on molecular systems in gas and condensed phases. *J. Chem. Phys.* **127** (2007).

7 Krack, M. Pseudopotentials for H to Kr optimized for gradient-corrected exchange-correlation functionals. *Theor. Chem. Acc.* **114**, 145-152 (2005).

8 Shen, C., Kirchhof, R. & Bertram, F. in *J. Phys.: Conf. Ser.* 012047 (IOP Publishing).

9 Bu, W. & Vaknin, D. X-ray fluorescence spectroscopy from ions at charged vapor/water interfaces. *J. Appl. Phys.* **105** (2009).

10 Oliveira, O. N., Jr., Caseli, L. & Ariga, K. The past and the future of Langmuir and Langmuir–Blodgett films. *Chem. Rev.* **122**, 6459-6513 (2022).

11 Funes-Ardoiz, I. & Maseras, F. Oxidative coupling mechanisms: current state of understanding. *ACS Catal.* **8**, 1161-1172 (2018).

12 Kong, Y. *et al.* Bridging the gap between reality and ideality of graphdiyne: the advances of synthetic methodology. *Chem* **6**, 1933-1951 (2020).

13 Fomina, L., Vazquez, B., Tkatchouk, E. & Fomine, S. The Glaser reaction mechanism. A DFT study. *Tetrahedron* **58**, 6741-6747 (2002).

14 Maity, S. & Patwari, G. N. Hydrogen bonding to multifunctional molecules: spectroscopic and ab initio investigation of water complexes of fluorophenylacetylenes. *J. Phys. Chem. A* **113**, 1760-1769 (2009).

15 Alwis, K. H. & Mucalo, M. R. In situ IR study of the anodic polarization of gold electrodes in polar aprotic solvents: DMSO and DMF solutions of cyanate, thiocyanate and selenocyanate ions. *J. Electrochem. Soc.* **161**, H738 (2014).

16 Zaba, T. *et al.* Formation of highly ordered self-assembled monolayers of alkynes on Au (111)

substrate. *J. Am. Chem. Soc.* **136**, 11918-11921 (2014).

17 Morsch, S., Lyon, S., Edmondson, S. & Gibbon, S. Reflectance in AFM-IR: implications for interpretation and remote analysis of the buried interface. *Anal. Chem.* **92**, 8117-8124 (2020).

18 Zhang, S., Liu, H., Huang, C., Cui, G. & Li, Y. Bulk graphdiyne powder applied for highly efficient lithium storage. *Chem. Commun.* **51**, 1834-1837 (2015).

19 Matsuoka, R. *et al.* Crystalline graphdiyne nanosheets produced at a gas/liquid or liquid/liquid interface. *J. Am. Chem. Soc.* **139**, 3145-3152 (2017).

20 Li, J. *et al.* A deprotection-free method for high-yield synthesis of graphdiyne powder with in situ formed CuO nanoparticles. *Angew. Chem. Int. Ed.* **61**, e202210242 (2022).

21 Kulkarni, R. *et al.* Direct growth of crystalline triazine-based graphdiyne using surface-assisted deprotection–polymerisation. *Chem. Sci.* **12**, 12661-12666 (2021).

22 Yang, L. *et al.* In situ deprotection-free synthesis of silver/graphdiyne with a high Raman sensing effect for detection of polychlorophenols and microplastics. *Inorg. Chem.* **63**, 21679-21686 (2024).

23 Hui, L. *et al.* Highly efficient and selective generation of ammonia and hydrogen on a graphdiyne-based catalyst. *J. Am. Chem. Soc.* **141**, 10677-10683 (2019).

24 Liu, R. *et al.* Chemical vapor deposition growth of linked carbon monolayers with acetylenic scaffoldings on silver foil. *Adv. Mater.* **29**, 1604665 (2017).

25 Zuo, Z. *et al.* A facile approach for graphdiyne preparation under atmosphere for an advanced battery anode. *Chem. Commun.* **53**, 8074-8077 (2017).

26 Li, G. *et al.* Architecture of graphdiyne nanoscale films. *Chem. Comm.* **46**, 3256-3258 (2010).

27 Yue, Y. *et al.* Conductive metallophthalocyanine framework films with high carrier mobility as efficient chemiresistors. *Angew. Chem. Int. Ed.* **60**, 10806-10813 (2021).

28 Yang, X. *et al.* Mesoporous polyimide-linked covalent organic framework with multiple redox-active sites for high-performance cathodic Li storage. *Angew. Chem. Int. Ed.* **61**, e202207043 (2022).

29 Wang, L. *et al.* A highly soluble, crystalline covalent organic framework compatible with device implementation. *Chem. Sci.* **10**, 1023-1028 (2019).

30 Li, T. *et al.* A two-dimensional semiconducting covalent organic framework with nickel(ii) coordination for high capacitive performance. *J. Mater. Chem. A* **7**, 19676-19681 (2019).

31 Lakshmi, V. *et al.* A two-dimensional poly(azatriangulene) covalent organic framework with semiconducting and paramagnetic states. *J. Am. Chem. Soc.* **142**, 2155-2160 (2020).

32 Jhulki, S. *et al.* Solution-processable, crystalline π-conjugated two-dimensional polymers with high charge carrier mobility. *Chem* **6**, 2035-2045 (2020).

33 Wang, R. *et al.* Highly conductive covalent–organic framework films. *Small* **20**, 2306634 (2024).

34 Wu, Y., Yan, D., Zhang, Z., Matsushita, M. M. & Awaga, K. Electron highways into nanochannels of covalent organic frameworks for high electrical conductivity and energy storage. *ACS Appl. Mater. Interfaces* **11**, 7661-7665 (2019).

35 Xu, X. *et al.* Janus dione-based conjugated covalent organic frameworks with high conductivity as superior cathode materials. *J. Am. Chem. Soc.* **145**, 1022-1030 (2023).

36 Yue, Y., Li, H., Chen, H. & Huang, N. Piperazine-linked covalent organic frameworks with high electrical conductivity. *J. Am. Chem. Soc.* **144**, 2873-2878 (2022).

37 Zhu, Y. *et al.* Construction of interlayer conjugated links in 2D covalent organic frameworks via topological polymerization. *J. Am. Chem. Soc.* **143**, 7897-7902 (2021).

38 Li, J. *et al.* Observing proton–electron mixed conductivity in graphdiyne. *Adv. Mater.* **36**, 2400950 (2024).

39 Qian, X. *et al.* Construction of graphdiyne nanowires with high-conductivity and mobility. *Dalton Trans.* **41**, 730-733 (2012).
